# Role of Severe Acute Respiratory Syndrome Coronavirus Viroporins E, 3a, and 8a in Replication and Pathogenesis

**DOI:** 10.1128/mBio.02325-17

**Published:** 2018-05-22

**Authors:** Carlos Castaño-Rodriguez, Jose M. Honrubia, Javier Gutiérrez-Álvarez, Marta L. DeDiego, Jose L. Nieto-Torres, Jose M. Jimenez-Guardeño, Jose A. Regla-Nava, Raul Fernandez-Delgado, Carmina Verdia-Báguena, Maria Queralt-Martín, Grazyna Kochan, Stanley Perlman, Vicente M. Aguilella, Isabel Sola, Luis Enjuanes

**Affiliations:** a Department of Molecular and Cell Biology, Centro Nacional de Biotecnología (CNB-CSIC), Campus Universidad Autónoma de Madrid, Madrid, Spain; b Eunice Kennedy Shriver NICHD, NIH, Bethesda, Maryland, USA; c Immunomodulation Group, Navarrabiomed-Biomedical Research Centre, IdISNA, Pamplona, Navarra, Spain; d Department of Microbiology, University of Iowa, Iowa City, Iowa, USA; e Department of Physics, Laboratory of Molecular Biophysics, Universitat Jaume I, Castelló, Spain; Vanderbilt University Medical Center

**Keywords:** coronavirus, PBM, PDZ, SARS-CoV, viroporins

## Abstract

Viroporins are viral proteins with ion channel (IC) activity that play an important role in several processes, including virus replication and pathogenesis. While many coronaviruses (CoVs) encode two viroporins, severe acute respiratory syndrome CoV (SARS-CoV) encodes three: proteins 3a, E, and 8a. Additionally, proteins 3a and E have a PDZ-binding motif (PBM), which can potentially bind over 400 cellular proteins which contain a PDZ domain, making them potentially important for the control of cell function. In the present work, a comparative study of the functional motifs included within the SARS-CoV viroporins was performed, mostly focusing on the roles of the IC and PBM of E and 3a proteins. Our results showed that the full-length E and 3a proteins were required for maximal SARS-CoV replication and virulence, whereas viroporin 8a had only a minor impact on these activities. A virus missing both the E and 3a proteins was not viable, whereas the presence of either protein with a functional PBM restored virus viability. E protein IC activity and the presence of its PBM were necessary for virulence in mice. In contrast, the presence or absence of the homologous motifs in protein 3a did not influence virus pathogenicity. Therefore, dominance of the IC and PBM of protein E over those of protein 3a was demonstrated in the induction of pathogenesis in mice.

## IMPORTANCE

Collectively, these results demonstrate key roles for the ion channel and PBM domains in optimal virus replication and pathogenesis and suggest that the viral viroporins and PBMs are suitable targets for antiviral therapy and for mutation in attenuated SARS-CoV vaccines.

## INTRODUCTION

Coronaviruses (CoVs) are pathogens responsible for a wide range of existing and emerging diseases in humans and domestic or companion animals (1). A CoV causing the severe acute respiratory syndrome (SARS-CoV) was identified in Southeast China in 2002 and rapidly spread worldwide to more than 30 countries within 6 months, infecting more than 8,000 people, with mortality in approximately 10% of the cases (2, 3). While SARS-CoV has not since reappeared in humans, other CoVs, including ones similar to SARS-CoV, are widely disseminated among bats circulating all over the world, making future outbreaks possible (4–6). In fact, a novel CoV, the Middle East respiratory syndrome coronavirus (MERS-CoV), was identified in September 2012 in two human patients with severe respiratory disease in Saudi Arabia (7, 8); since then, the WHO has reported 2,144 laboratory-confirmed cases and at least 750 deaths (as of 29 March 2018) (http://www.who.int/emergencies/mers-cov/en/). These data indicate that emergence of other highly pathogenic CoVs is likely and thus that the study of the virus-host interaction is essential to develop antiviral therapies and safe vaccines.

Viroporins constitute a large class of multifunctional viral proteins with ion channel (IC) activity that are widely distributed among different viral families (9); highly pathogenic human viruses such as human immunodeficiency virus 1 (HIV-1), hepatitis C virus (HCV), influenza A virus (IAV), rotavirus (RV), enterovirus, and CoVs such as SARS-CoV and MERS-CoV encode them (10–16). Viroporins promote several steps of the virus replication cycle, including entry, genome replication, morphogenesis, and release from the infected cell (17, 18). Several viroporins have important roles in viral pathogenesis, promoting ion imbalances within cells (13, 19–21) or disrupting cellular pathways through protein-protein interactions (22). Given their potential as antiviral targets, there is substantial interest in the study of these proteins (18, 23).

Several CoVs, such as MERS-CoV, HCoV-229E, HCoV-OC43, and porcine epidemic diarrhea virus (PEDV), encode two viroporins (24–26), but, remarkably, SARS-CoV encodes three: proteins 3a, E, and 8a (14, 27, 28). The 3a protein is 274 amino acids (aa) in length with three transmembrane domains (TMDs). It is the largest SARS-CoV accessory protein and is likely involved in virus release (27) and pathogenesis (29). It causes membrane rearrangements in infected cells, leading to an increase in levels of intracellular vesicles that may facilitate nonlytic release of viral particles (30). Furthermore, it colocalizes with M protein, which, together with E protein, is essential for virus assembly, supporting the notion that the 3a protein is important for SARS-CoV assembly or budding (31, 32). However, most studies have been based on 3a overexpression and little is known about the relevance of this protein in the context of natural infection.

SARS-CoV E protein is an integral membrane protein of 76 aa with only one TMD. The residues responsible for E protein IC activity have been previously identified (33, 34). E protein IC activity is important for SARS-CoV fitness and pathogenesis, since both were diminished in its absence (34).

The 8a protein found in SARS-CoV-infected human cells resulted from a 29-nucleotide (nt) deletion in open reading frame 8 (ORF8) that occurred after the virus crossed species to infect humans (35). ORF8 genes encode two proteins, ORF8a and ORF8b, which represent proteins of 39 and 84 aa, respectively. Overexpression assays showed that ORF8a induces apoptosis through a mitochondrion-dependent pathway (36) and has IC activity (28).

Viroporins and cellular IC proteins often rely on protein-protein interactions for clustering of ICs at proper locations in the cell (37–39). These interactions are mediated between PDZ domains and PDZ-binding motifs (PBMs), peptide sequences that are most frequently located at the C terminus of the IC proteins (40, 41). PDZ domains are protein recognition sequences, 80 to 90 aa in length, and constitute a large family of globular domains found in prokaryotes and eukaryotes. There are more than 400 cellular protein isoforms containing a PDZ domain in the human proteome (42). The PBM core sequence includes 4-aa residues, numbered from the C terminus (p0), which is always hydrophobic, to the N terminus (p-1, p-2, and p-3). There are three classes of PBMs, depending on the identity of residue p-2: class I for Thr/Ser, class II for any hydrophobic residue, and class III for Glu/Asp. Protein-protein interactions involving PDZ domains modulate cellular pathways important for viral replication, dissemination in the host, and pathogenesis (43). Furthermore, some PDZs also bind PBMs located in the internal region of proteins or lipids (44, 45). Of the three SARS-CoV viroporins, both proteins 3a and E have a class II PBM at their C terminus; while the PBM of E protein is involved in pathogenesis (46, 47), the role of the PBM of protein 3a, and of similar motifs present in other CoV proteins such as MERS-CoV proteins E and 5, remains unknown.

In the present work, using mutational analysis, we showed that only the 3a and E proteins were clearly involved in SARS-CoV replication and virulence. Neither single deletion of the IC activity or of PBM from protein 3a diminished SARS-CoV replication and virulence, in contrast with E protein, which required both for virulence (34, 46). Protein 3a IC activity was characterized in planar lipid bilayers, showing that it forms non-voltage-gated ion channels. Furthermore, we identified residues located in TMD2 and TMD3 of the 3a protein that were involved in its IC activity. The potential interdependence of the three viroporins described in SARS-CoV was studied by deletion of single viroporins or of different combinations of two viroporins. The variant missing both the 3a and E proteins was not viable, indicating that the presence of at least one of the proteins is essential for virus viability. Furthermore, it was shown that either protein should maintain its PBM to compensate for the absence of the other full-length protein. These results suggest that PBMs interact with cellular proteins with PDZ domains to change cell metabolism, enhancing virus replication or pathogenicity. Identification and inhibition of specific cellular pathways affected by these interactions may be crucial for the identification of new antiviral strategies.

## RESULTS

### SARS-CoV viroporins E and 3a were required for efficient replication *in vitro* and *in vivo*.

**FIG 1:**
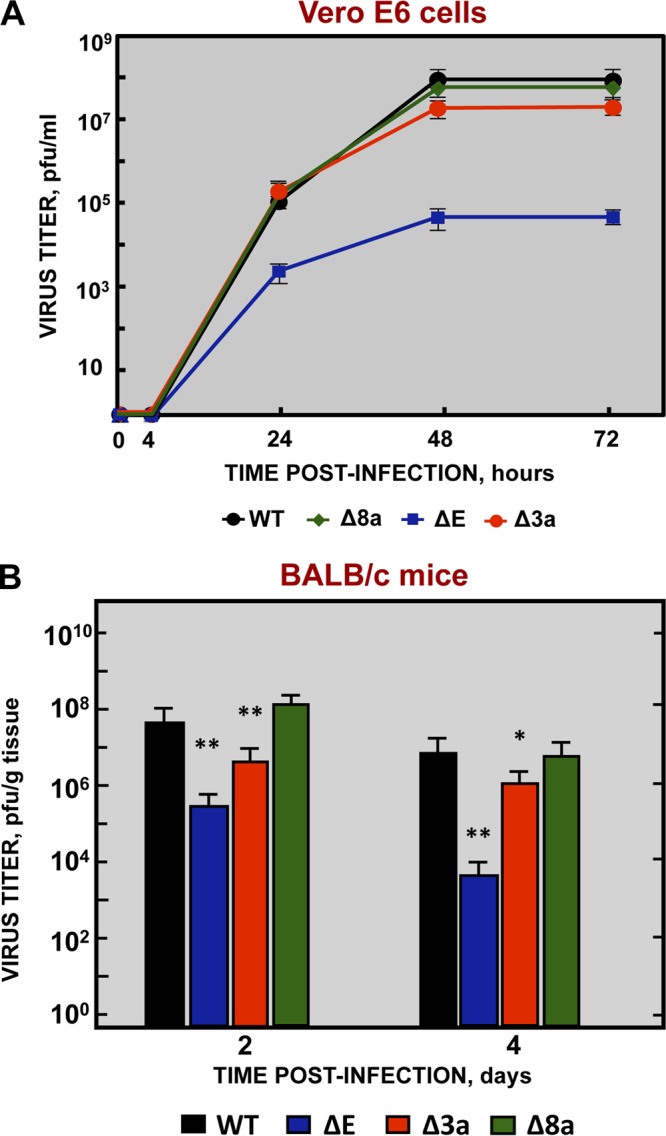
Growth kinetics of SARS-CoV viroporin-defective mutants. (A) Subconfluent monolayers of Vero E6 cells were infected with wild-type (WT) (black filled circles), ΔE (blue filled squares), Δ3a (red filled circles), and Δ8a (green filled diamonds) SARS-CoV at a MOI of 0.001. Culture supernatants were collected at 4, 24, 48, and 72 hpi and titrated by plaque assay. The results are representative of three replicate experiments. (B) Groups of six 16-week-old BALB/c mice were infected with 100,000 PFU of either the parental virus (WT, black columns) or genetically engineered viruses lacking E protein (ΔE, blue columns), 3a protein (Δ3a, red columns), or 8a protein (Δ8a, green columns). At 2 and 4 dpi, 3 mice from each group were sacrificed to determine lung virus titers. Data summarize two replicate experiments. Data represent means ± standard deviations (SD). *, *P* value <0.1; **, *P* value <0.01; ***, *P* value <0.001.

To study the role of the SARS-CoV viroporins in virus replication and virulence, three mutant viruses, each lacking one gene (recombinant SARS [rSARS]-CoV-MA15-Δ3a, -ΔE, and -Δ8a), were engineered from a mouse-adapted infectious cDNA clone (MA15) (48, 49). Analysis of the growth kinetics of each mutant in Vero E6 cell supernatants (Fig. 1A) was used to determine their requirement for replication. Cell-associated virus was also analyzed at 24 and 48 h postinfection (hpi), showing results similar to those observed for the released virus (see Fig. S1 in the supplemental material). The Δ3a and ΔE mutants grew to lower titers than the parental wild-type (wt) virus (Fig. 1A). However, while the ΔE mutant showed 100-fold-lower titers (around 8 × 10^5^ PFU/ml), Δ3a titers decreased slightly (3-fold) (3 × 10^7^ PFU/ml). These results show that both proteins were required for optimal virus replication in cell culture. In contrast, the Δ8a virus reached peak titers (9 × 10^7^ PFU/ml) similar to those observed for the parental virus.

10.1128/mBio.02325-17.1 FIG S1  Analysis of cell-associated virus and released virus. Subconfluent monolayers of Vero E6 cells were infected with SARS-CoV wt, ΔE, Δ3a, and Δ8a viruses at a MOI of 0.001. Viral titers were analyzed at 24 hpi (A) and 48 hpi **(**B). Released virus (blue columns) was obtained from cell culture supernatants, and cell-associated virus (red columns) was recovered after cells were disrupted by four freeze-thaw cycles. Then, viruses were titrated by plaque assay. The results are representative of three replicate experiments. Download FIG S1, TIF file, 0.1 MB. Copyright © 2018 Castaño-Rodriguez et al. 2018 Castaño-Rodriguez et al. This content is distributed under the terms of the Creative Commons Attribution 4.0 International license.

To evaluate the requirement for protein 3a, E, or 8a for optimal virus growth *in vivo*, BALB/c mice were infected either with rSARS-CoV-MA15 or with each of the viroporin deletion mutants SARS-CoV-MA15-Δ3a, -ΔE, and -Δ8a, and viral titers in lungs were determined at 2 and 4 days postinfection (dpi) (Fig. 1B). The highest titers were reached at 2 dpi, and the titers decreased in all cases by between 5-fold and 40-fold at 4 dpi, with the parental and Δ8a viruses achieving the highest titers in lung tissue (around 10^8^ PFU/g at 2 dpi and 2 × 10^7^ PFU/g at 4 dpi). However, compared to the parental virus, titers were reduced by 1 and 2 log units in the case of Δ3a and ΔE virus, respectively. Interestingly, the decrease in virus titers after SARS-CoV-MA15-Δ3a infection was greater *in vivo* than *in vitro*. Thus, proteins E and 3a were shown to be critical for both *in vitro* and *in vivo* virus replication.

### SARS-CoV viroporins E and 3a were both associated with virulence in a mouse model.

**FIG 2:**
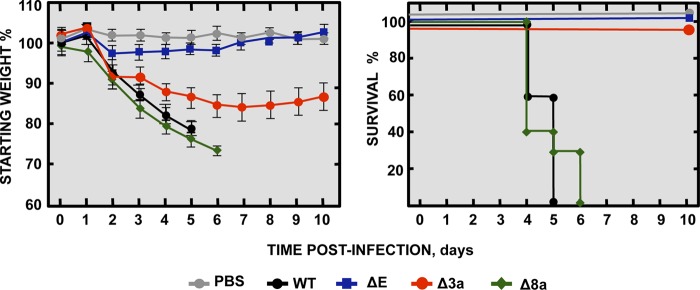
Virulence of SARS-CoV viroporin-defective mutants. Groups of five 16-week-old BALB/c mice were subjected to mock infection (PBS, gray filled circles) or infected with 100,000 PFU of either the parental virus (wt, black filled circles) or genetically engineered viruses missing E protein (ΔE, blue filled squares), 3a protein (Δ3a, red filled circles), or 8a protein (Δ8a, green filled diamonds). Mean levels of weight loss (left graph) and survival (right graph) through 10 dpi are shown for each group. Data summarize two replicate experiments with equivalent results. Error bars represent the standard deviations of mouse weight data.

To evaluate the relevance of SARS-CoV E, 3a, and 8a viroporins for virulence, BALB/c mice were either subjected to mock infection or infected with parental rSARS-CoV-MA15 or with one of the deletion mutants rSARS-CoV-MA15-Δ3a, -ΔE, and -Δ8a. Clinical disease and survival were monitored through 10 dpi (Fig. 2). Mice infected with viruses lacking either E protein or 3a protein recovered from infection with 100% survival, although mice infected with the Δ3a virus showed mild disease symptoms. In contrast, mice infected with the parental virus or the Δ8a virus developed manifestations of serious disease (lethargy and ruffled fur) starting from 2 dpi. These mice all died by 6 dpi, clearly showing that both E and 3a proteins were involved in SARS-CoV virulence in the mouse model, while 8a did not seem to play a major role.

### Characterization of the IC activity of protein 3a in planar lipid bilayers.

**FIG 3:**
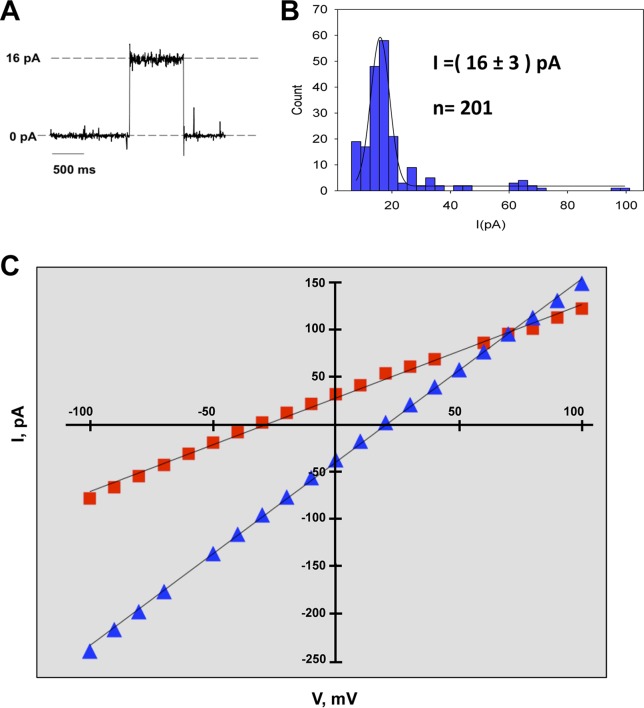
Characterization of the SARS-CoV 3a protein ion channel. (A) Recording of a single-channel insertion of SARS-CoV 3a protein. (B) Histogram of current jump amplitude (right) at +100 mV in 500 mM KCl, composed of values from 201 recording events. (C) SARS-CoV 3a protein voltage-independent ion channel. The 3a protein showed a linear current-voltage relationship. Displayed data correspond to representative I-V plots from reversal potential experiments performed with 500/50 mM solutions of monovalent (NaCl, red filled squares) and divalent (CaCl_2_, blue filled triangles) cations. Each experiment was performed at least three times; the lines represent linear regression fits of data points.

The IC activity of protein E is required for SARS-CoV replication and virulence (34). However, as the relevance of the IC activity of the 3a protein was not known, we studied 3a protein in planar lipid bilayers and identified the amino acids involved in ion conductance. This system was used because its high sensitivity allows the detection of electric currents of a single ion channel (50). To this end, a baculovirus was engineered to express the parental 3a protein in Sf-9 cells. Conductance of purified protein 3a was evaluated in the presence of KCl in planar lipid bilayers with a biologically relevant mix of 1,2-dioleoyl-*sn*-glycero-3-phosphocholine (DOPC)/1,2-dioleoyl-*sn*-glycero-3-phospho-l-serine (DOPS)/1,2-dioleoyl-*sn*-glycero-3-phosphoethanolamine (DOPE) with ratios of 3:1:1 (wt/wt), which is a composition similar to that of intracellular organelle membranes, such as the endoplasmic reticulum (ER)-Golgi intermediate compartment (ERGIC). Single-channel conductance was estimated from a statistical analysis of the current jump amplitudes. This procedure allows a reliable estimate of the most probable value of current change every time a new channel is inserted or disappears. Although several channels were being inserted, the magnitude of the current through a single channel could be discriminated. Current jumps corresponding to 201 independent events were measured under conditions of an applied voltage of +100 mV. Histograms of the current jump amplitudes of the recorded traces showed that the most frequent events corresponded to single-channel conductance of 16 pA (Fig. 3A and B). Simultaneous bursts of two or three 3a ion channels were also observed, although with much lower frequency (Fig. 3B).

To test if 3a behaves as a voltage-gated IC, its activity was also measured in planar lipid bilayers under conditions of different voltages in the presence of monovalent (NaCl) and divalent (CaCl_2_) cations (Fig. 3C). In all cases, a linear current-voltage (I-V) relationship was obtained, demonstrating that the channel displayed resistance (ohmic) behavior for both positive and negative potential. These results indicate that the protein 3a IC was neither open nor closed at specific electric potentials.

Measurement of the reversal potential (*E*_rev_) of an ion channel, which is defined as the voltage that needs to be applied to yield zero electric current when there is an ion concentration gradient across the membrane, is the method of choice to quantify ion selectivity. Determination of the sign of the *E*_rev_ provides a quick estimation of the channel selectivity, that is, of its preference for cations or anions (51, 52). By comparing the measured *E*_rev_ to the theoretical *E*_rev_ that would be obtained in the case of a neutral pore (i.e., representing only the difference between cation and anion intrinsic mobilities), the selectivity of the ion channel can be inferred. The theoretical *E*_rev_ is calculated using the Goldman-Hodgkin-Katz (GHK) equation, replacing the permeability ratio P_+_/P_−_ by the solution diffusion coefficient ratio D_+_/D_−_ (53). Higher, lower, or similar measured *E*_rev_ values indicate anion selectivity, cation selectivity, or no selectivity, respectively. Interestingly, in the presence of monovalent ions (Na^+^ and K^+^), the protein 3a IC showed weak cation selectivity. However, in the presence of Ca^++^, the channel behaved as a neutral channel with no preference for anions or cations (Table 1). Taken together, these results indicated that at least Na^+^, K^+^, and Ca^++^ were conducted through the 3a protein IC.

**TABLE 1:** Results of protein 3a reversal potential experiments performed with 500/50 mM salt solutions

Ion solution	*E*_rev_ (mV)^a^	
	Experimental	Reference
NaCl	−19.1 ± 14.8	+8.86
KCl	−13.0 ± 4.0	+0.73
CaCl_2_	+18.8 ± 6.0	+20.3

a Experimental reversal potential (*E*_rev_) values represent the averages of results from at least 7 independent experiments. Reference *E*_rev_ values represent theoretical values for a neutral pore.

### Identification of amino acids involved in protein 3a IC activity.

**FIG 4:**
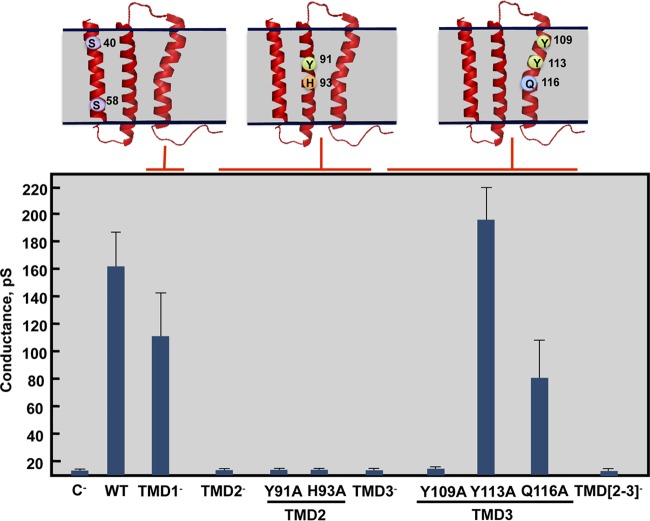
Effect of mutations on the ion channel activity of SARS-CoV 3a protein. Recombinant 3a protein variants were reconstituted in artificial lipid bilayers, and their IC activity was tested in 500 mM KCl solutions. Mean conductance values were measured for variants showing IC activity. Negative control (C^−^) data indicate conductance values obtained in the absence of any protein; error bars represent the standard deviations of data obtained in at least 100 independent measurements.

In order to identify the amino acids necessary for protein 3a IC activity, a set of recombinant baculoviruses (rBV) expressing mutated 3a proteins was engineered. Amino acid substitutions were created to disrupt the IC activity of 3a with minimal impact on its three-dimensional structure, mutating residues predicted to face the lumen of the pore. As the 3a protein structure has not yet been experimentally determined, *in silico* models were used to select the residues potentially facing the lumen of the pore (54, 55). As the 3a protein has three TMDs, mutants with changes to a single TMD (TMD1^−^, TMD2^−^, or TMD3^−^) or to two TMDs (TMD[2,3]^−^) were engineered (Table 2). Mutant 3a proteins were expressed in insect cells and purified, and their IC activity was evaluated in planar lipid bilayers (Fig. 4). The TMD1^−^ mutant retained IC activity, but the TMD2^−^, TMD3^−^, and TMD[2,3]^−^ variants did not, consistent with the importance of TMD2 and TMD3, as predicted by *in silico* models (54, 55).

**TABLE 2:** 3a protein ion channel mutations

Mutant	Mutations
TMD1^−^	S40A, S58A
TMD2^−^	Y91A, H93A
TMD3^−^	Y109A, Y113A, Q116A
TMD[2,3]^−^	Y91A, H93A, Y109A, Y113A, Q116A

To resolve the exact residues necessary for protein 3a IC activity, a complementary set of baculoviruses incorporating single amino acid substitutions within mutants TMD2^−^ (Y91A and H93A) and TMD3^−^ (Y109A, Y113A, and Q116A) was generated. These mutant proteins were expressed and purified, and their IC activity was evaluated. TMD2 point mutants Y91A and H93A and TMD3 point mutant Y109A completely abrogated protein 3a IC activity, whereas TMD3 point mutants Y113 and Q116 showed conductance that was equivalent to and only moderately decreased from that seen with the wt protein, respectively. Therefore, these results identified 3 amino acids that could be mutated to eliminate protein 3a IC activity.

### Protein 3a IC activity was not required for SARS-CoV replication and virulence.

**FIG 5:**
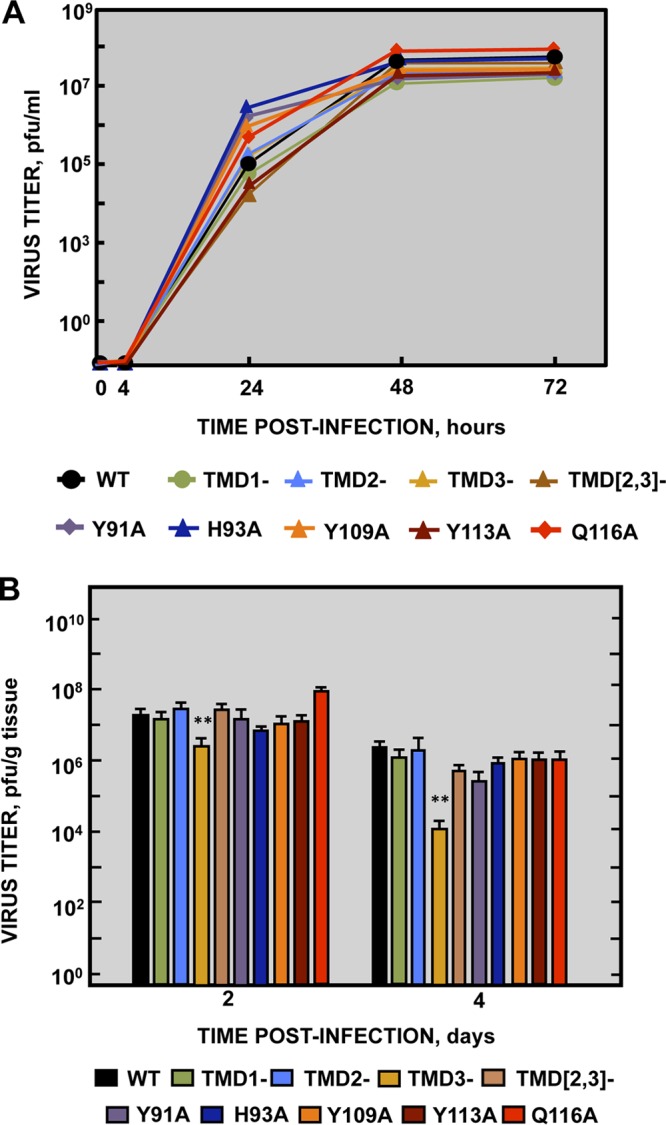
Growth kinetics of SARS-CoV mutants targeting 3a protein ion channel activity. (A) Subconfluent monolayers of Vero E6 cells were infected at a MOI of 0.001 with wild-type SARS-CoV (WT, black filled circles) or with variants with mutations affecting 3a protein TMD1 (TMD1-, light green filled circles), TMD2 (TMD2-, light blue filled triangles), TMD3 (TMD3-, ochre filled triangles), or both TMD2 and TMD3 (TMD[2,3]-, light brown filled triangles) or residue Y91 (Y91A, purple filled diamonds), residue H93 (H93A, deep blue filled diamonds), residue Y109 (Y109A, orange filled diamonds), residue Y113 (Y113A, dark brown filled diamonds), or residue Q116 (Q116A, red filled diamonds). Culture supernatants collected at 4, 24, 48, and 72 hpi were titrated by plaque assay. Results are representative of three replicate experiments. For the sake of clarity, SD data are not shown but the values were, in all cases, lower than 5%. (B) Groups of six 16-week-old BALB/c mice were infected with 100,000 PFU of either the parental virus (WT, black columns), or mutants TMD1^−^ (light green columns), TMD2^−^ (light blue columns), TMD3^−^ (ochre columns), TMD[2,3]^−^ (light brown columns), Y91A (purple columns), H93A (deep blue columns), Y109A (orange columns), Y113A (dark brown columns), and Q116A (red columns). At 2 and 4 dpi, 3 mice from each group were sacrificed to determine virus titers. Data summarize two replicate experiments. Data represent means ± SD. *, *P* value <0.1; **, *P* value <0.01; ***, *P* value <0.001.

To study the relevance of protein 3a IC activity in virus replication and virulence, the following collection of full-length rSARS-CoVs with and without protein 3a IC activity was generated by introducing specific mutations into the 3a gene: rSARS-CoV-MA15-3a-TMD1^−^, -TMD2^−^, -TMD3^−^, -TMD[2,3]^−^, -Y91A, -H93A, -Y109A, -Y113A, and -Q116A. All these viruses were similar with respect to growth kinetics in Vero E6 cells (Fig. 5A), indicating that replication was not significantly affected by altered protein 3a IC activity.

The requirement for protein 3a IC activity *in vivo* was also studied by measuring the titers of 3a IC mutants in the lungs of infected BALB/c mice at 2 and 4 dpi (Fig. 5B). Peak titers were reached at 2 dpi and had decreased by around 1 log unit at 4 dpi in all cases. Every mutant showed replication levels similar to those seen with the wt strain (around 1 × 10^7^ PFU/g of lung tissue at 2 dpi and 4 × 10^6^ PFU/g at 4 dpi), with the exception of TMD3^−^, which had titers at least 1 log unit lower than those seen with the rest of the viruses (3 × 10^6^ PFU/g at 2 dpi and 1 × 10^4^ PFU/g at 4 dpi) (Fig. 5B). These results indicated that protein 3a IC activity was not essential for SARS-CoV replication in mouse lungs.

**FIG 6:**
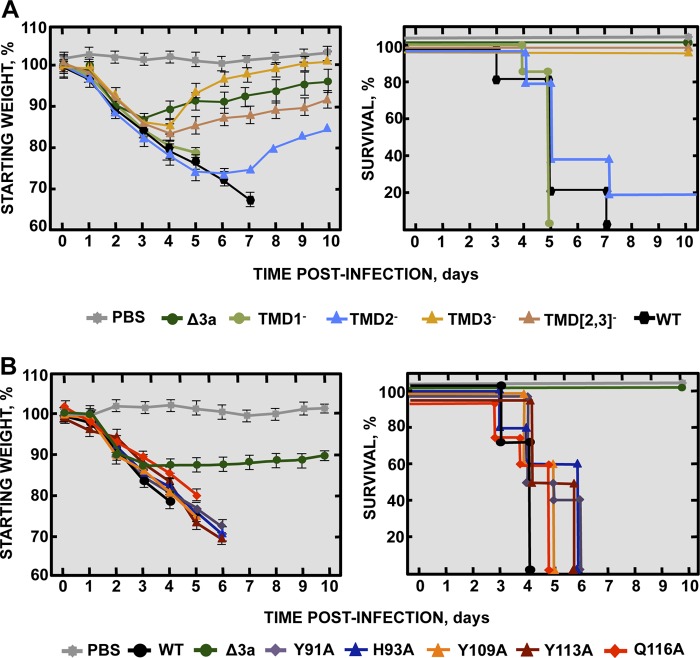
Virulence of SARS-CoV 3a ion channel mutants. Groups of five 16-week-old BALB/c mice were subjected to mock infection (PBS, gray filled squares) or infected with 100,000 PFU of the parental virus (WT, black hexagons) or with genetically engineered mutants lacking the 3a protein (Δ3a, dark green filled circles), and (A) mutants with altered TMD1 (TMD1^−^, light green filled circles), TMD2 (TMD2^−^, light blue filled diamonds), TMD3 (TMD3^−^, ochre filled diamonds), or both TMD2 and TMD3 (TMD[2,3]^−^, brown filled diamonds). (B) Additional comparisons with 3a mutants Y91A (purple filled diamonds), H93A (deep blue filled triangles), Y109A (orange filled triangles), Y113A (brown filled triangles), or Q116A (red filled diamonds) were performed. All mice were evaluated for weight loss (left) and survival (right) through 10 dpi. Data summarize two replicate experiments with equivalent results. Error bars represent the standard deviations for mouse weight.

The requirement of protein 3a IC activity for SARS-CoV virulence was studied in two independent experiments. In the first, BALB/c mice were infected with rSARS-CoV-MA15 (virulent virus control), rSARS-CoV-MA15-Δ3a (attenuated virus control), or one of the rSARS-CoV mutants TMD1^−^, TMD2^−^, TMD3^−^, and TMD[2,3]^−^ (Fig. 6A), with clinical disease and survival evaluated for 10 days. All mice infected with the parental virus or the TMD1^−^ mutant showed disease symptoms starting at 2 dpi, and all died at 5 or 7 dpi, respectively. Mice infected with the TMD2^−^ mutant showed acute disease starting at 2 dpi, and 80% of the mice had died by between 4 and 7 dpi. In contrast, mice infected with either TMD3^−^ or TMD[2,3]^−^ mutants recovered from the disease with 100% survival, similarly to mice infected with the attenuated SARS-CoV-Δ3a variant (Fig. 6A). During the experiment, viruses were recovered from the lungs of moribund mice, and the 3a gene was sequenced. No compensatory mutations restoring IC activity were identified in any case. The results suggest that the TMD3^−^ and TMD[2,3]^−^ mutants were attenuated in an IC-independent manner, since the TMD2^−^ mutant was only marginally attenuated.

In the second virulence experiment, mice were infected with rSARS-CoV incorporating protein 3a point mutations (Y91A, H93A, Y109A, Y113A, and Q116A), with the parental and Δ3a variants serving as virulent and attenuated controls, respectively. All of the 3a point mutants caused severe disease with 100% mortality by 6 dpi, similarly to mice infected with the wt parent and in contrast to those infected with the Δ3a variant, which survived (Fig. 6B). No compensatory mutations restoring IC activity were identified in this experiment, further confirming that protein 3a IC activity was not essential for SARS-CoV virulence in the mouse model used.

### The PBM of protein 3a was not required for SARS-CoV replication and virulence.

**FIG 7:**
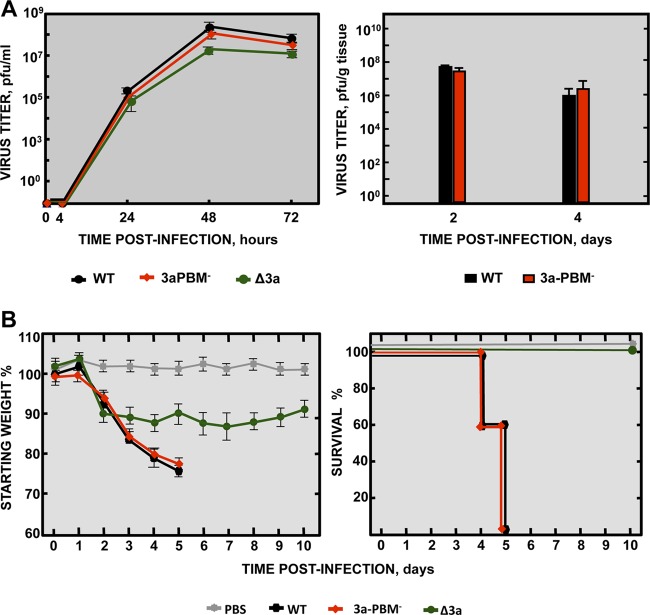
Requirement of the PBM of SARS-CoV 3a protein for replication and virulence. (A) (Left panel) Subconfluent monolayers of Vero E6 cells were infected with wild-type (WT; black filled circles), Δ3a (green filled circles), or 3a-PBM^−^ (red filled diamonds) SARS-CoV at a MOI of 0.001. Culture supernatants collected at 4, 24, 48, and 72 hpi were titrated by plaque assay. Results are representative of three replicate experiments. (Right panel) Groups of six 16-week-old BALB/c mice were infected with 100,000 PFU of the parental virus (WT, black columns) or of a SARS-CoV variant lacking the protein 3a PBM (3a-PBM^−^, red columns). At 2 and 4 dpi, 3 mice from each group were sacrificed to determine virus titers. Data summarize two replicate experiments. Data represent means ± SD. (B) Groups of five 16-week-old BALB/c mice were subjected to mock infection (PBS, gray filled squares) or infected with 100,000 PFU of the parental (wild-type) virus (WT; black filled hexagons) or of genetically engineered mutants lacking the 3a protein (Δ3a, green filled circles) or lacking the protein 3a PBM (3a-PBM^−^, red filled diamonds). Mean levels of weight loss (left graph) and survival (right graph) through 10 dpi are represented for each group. Data summarize two replicate experiments with equivalent results. Error bars represent the standard deviations of mouse weight data.

To analyze the requirement for the PBM of protein 3a for replication and virulence, a virus lacking a functional PBM in the 3a protein (3aPBM^−^) was engineered. As ORF3a partially overlaps ORF3b, the protein 3a PBM core sequence (SVPL) was disrupted with amino acid substitutions (GMSM), with codons carefully selected to ensure that protein ORF3b was not mutated. Growth of the 3aPBM^−^ mutant in Vero E6 cells and in the lungs of infected mice was the same as that seen with rSARS-CoV-MA15 (Fig. 7A). Also, the 3aPBM^−^ mutant was as pathogenic as the parental virus (Fig. 7B). These results indicated that, in the mouse model, replication and virus virulence were independent of the PBM of protein 3a.

### Simultaneous requirement of viroporins by SARS-CoV.

In order to study the interdependence of SARS-CoV viroporins, all possible combinations of single-, double-, and triple-deletion mutants were engineered (Table 3). All combinations were efficiently rescued, with the remarkable exceptions of the triple mutant [rSARS-CoV-MA15-Δ(3a,E,8a)] and the one lacking both E and 3a proteins [rSARS-CoV-MA15-Δ(3a,E)]. The ΔE virus and double-deletion mutants Δ(3a,8a) and Δ(E,8a) showed significantly reduced titers (1 × 10^6^, 2 × 10^6^, and 6 × 10^5^ PFU/ml, respectively) compared to the Δ3a and Δ8a mutants and the parental virus. Although a role for protein 8a was not described for virus replication, the titers of viruses missing 8a and 3a or E were significantly lower than the titers of Δ3a and ΔE viruses. Also, every one of the viable mutant viruses which lacked either 3a or E proteins showed plaque sizes that were significantly smaller than those seen with the wt and Δ8a viruses.

**TABLE 3:** Simultaneous requirements of viroporins by SARS-CoV

Virus name	SARS-CoV viroporin			Viral titer (PFU/ml)
	3a	E	8a	
SARS-CoV wt	**+**	**+**	**+**	(4.0 ± 1.2) × 10^7^
SARS-CoV Δ8a	**+**	**+**	**−**	(5.0 ± 2.1) × 10^7^
SARS-CoV Δ3a	**−**	**+**	**+**	(1.0 ± 1.9) × 10^7^
SARS-CoV ΔE	**+**	**−**	**+**	(1.0 ± 0.8) × 10^6^
SARS-CoV Δ[E, 8a]	**+**	**−**	**−**	(6.6 ± 1.4) × 10^5^
SARS-CoV Δ[3a, 8a]	**−**	**+**	**−**	(2.4 ± 1.1) × 10^6^
SARS-CoV Δ[3a, E]	**−**	**−**	**+**	<2.0 × 10^1^^a^
SARS-CoV Δ[3a, E, 8a]	**−**	**−**	**−**	<2.0 × 10^1^^a^

a Data were below the detection threshold.

The results showed that at least either the 3a protein or the E protein must be present for virus viability, which indicates that just one of these proteins could provide the activities required for virus growth.

### SARS-CoV 3a protein subcellular localization.

**FIG 8:**
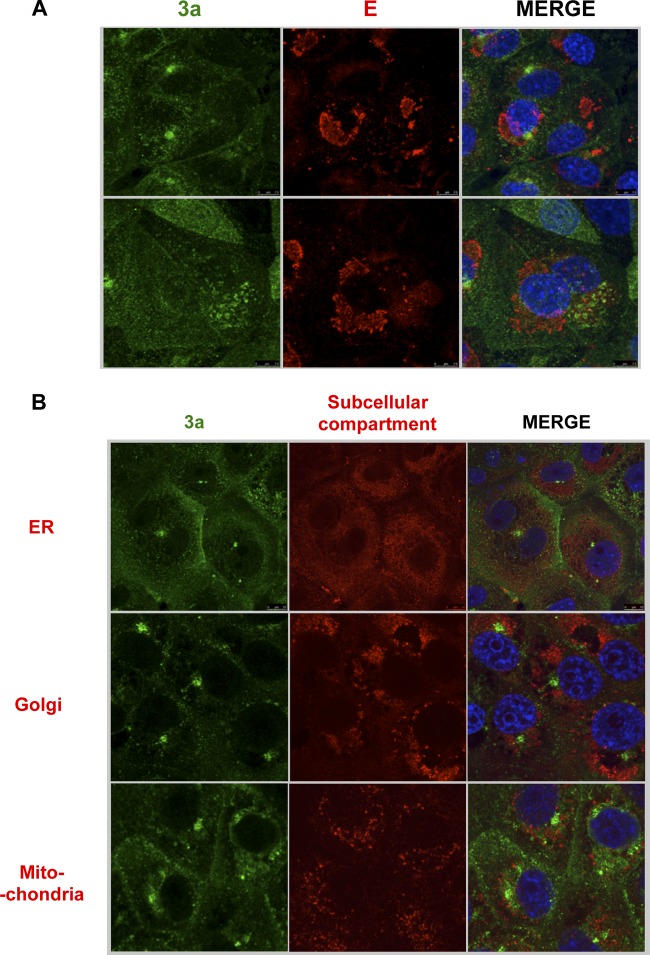
Analysis of the subcellular localization of SARS-CoV 3a and E proteins by immunofluorescence. Vero E6 cells were grown on coverslips and infected with rSARS-CoV at a MOI of 0.3 and were subsequently fixed with 4% paraformaldehyde at 24 hpi. (A) Cells were labeled with antibodies specific for 3a protein (green) or E protein (red). (B) Cells were labeled with antibodies specific for 3a protein (shown in green) or for PDI (ER marker), 58 K (Golgi marker), or aconitase 2 (mitochondrion marker) (shown in red). In all cases, nuclei were stained with DAPI (blue).

**FIG 9:**
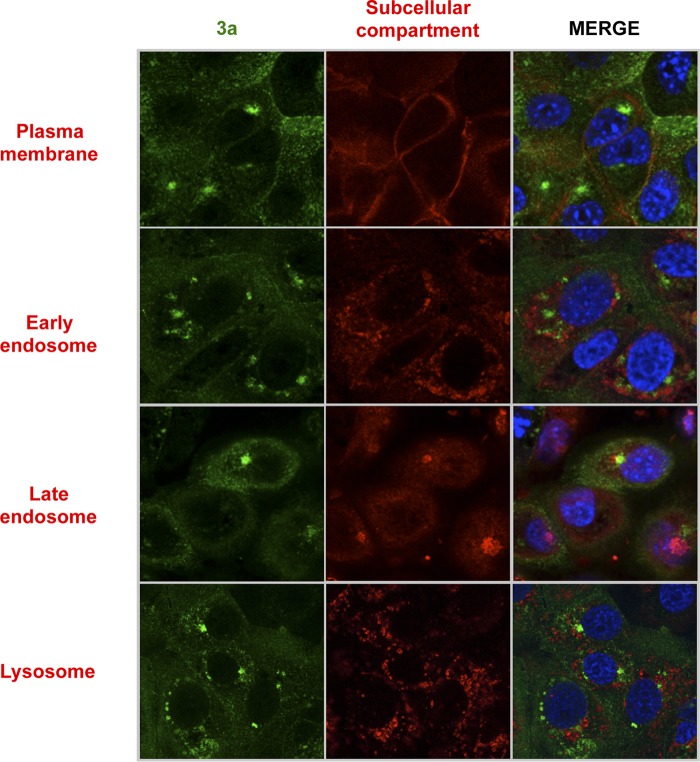
Analysis of the subcellular localization of SARS-CoV 3a by immunofluorescence. Vero E6 cells were grown on coverslips and infected with rSARS-CoV at a MOI of 0.3 and were fixed with 4% paraformaldehyde at 24 hpi. Cells were labeled with antibodies specific for 3a protein (shown in green) or for Na^+^/K^+^ ATPase (plasma membrane marker), rab5 (early endosome marker), rab7 (late endosome marker), and lamp-1 (lysosome marker) (shown in red). In all cases, nuclei were stained with DAPI (blue).

To begin to understand the nature of the replacement of E protein by 3a, or vice versa, in the replication of SARS-CoV, we assessed the cellular localization of each protein. The SARS-CoV E protein localizes to the ERGIC (56), but less is known about protein 3a localization (27). The potential colocalization of the 3a and E proteins was first studied by infecting Vero E6 cells with wt rSARS-CoV. Confocal microscopy analysis of rSARS-CoV-MA15 at 24 hpi showed that the proteins were located in different cell subcompartments during infection, as the Pearson’s coefficient value was below 0.6 (see Table S1 in the supplemental material) (57); protein 3a mainly accumulated at an unidentified perinuclear compartment different from the ERGIC (Fig. 8A). E protein IC activity promotes virulence by releasing calcium from the ERGIC, leading to inflammasome activation (34, 58). We showed that protein 3a IC activity is not involved in virus virulence. To extend these results, we next determined whether 3a was localized to any of the known cellular calcium reservoirs, i.e., the ER, the Golgi apparatus, and the mitochondria (59), using specific markers, including protein disulfide isomerase (PDI) (ER marker), 58 K (Golgi marker), and aconitase 2 (mitochondrion marker) (Fig. 8B). Also, other subcellular compartments were analyzed for colocalization with the 3a protein by the use of antibodies (Abs) against Na^+^/K^+^ ATPase (plasma membrane marker), Rab5 (early endosome marker), Rab7 (late endosome marker), and LAMP-1 (lysosome marker) (Fig. 9). Pearson’s coefficient was below 0.6 in all cases (Table S1). Overall, 3a protein did not localize at any of the main intracellular calcium storage locations, suggesting that it is not located at a site that facilitates increased cytosolic calcium levels. Collectively, these results suggest that the IC activity of proteins 3a and E, while shared by the two proteins, is not the function responsible for the replacement for the full-length E and 3a proteins.

10.1128/mBio.02325-17.5 TABLE S1  Pearson’s coefficient analysis. Download TABLE S1, DOCX file, 0.04 MB. Copyright © 2018 Castaño-Rodriguez et al. 2018 Castaño-Rodriguez et al. This content is distributed under the terms of the Creative Commons Attribution 4.0 International license.

### Identification of candidate motifs responsible for the replacement between E and 3a proteins.

**FIG 10:**
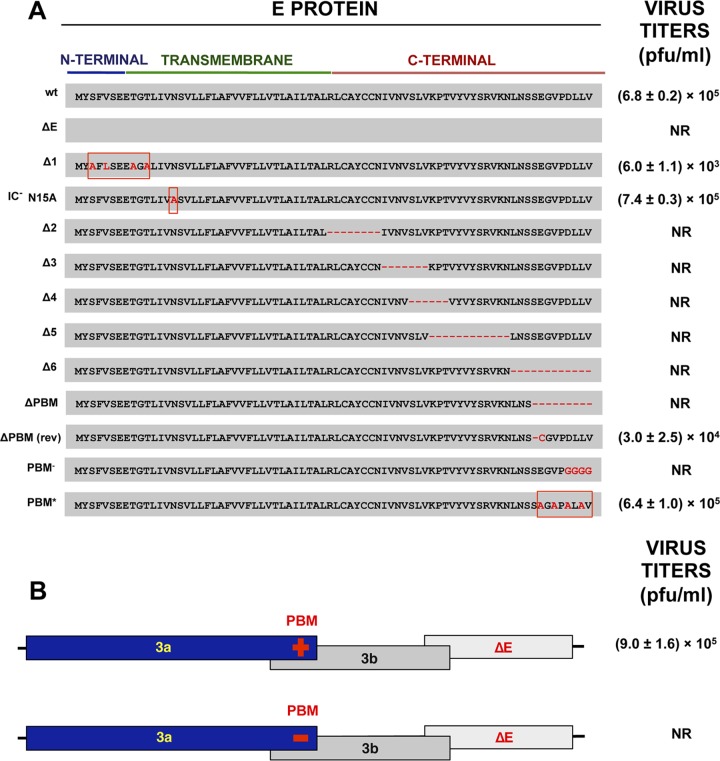
Mapping of the E protein domain required for the replacement of the 3a protein. (A) Viability of a set of SARS-CoV Δ3a mutants with added mutations within the E gene was evaluated in Vero E6 cells. Changes to the E protein included the following: alanine substitutions in the N-terminal domain [rSARS-CoV-MA15-(Δ3a,EΔ1)]; disruption of the TMD, abrogating E protein IC activity [(Δ3a,E-N15A)]; short in-frame deletions throughout the C-terminal domain [(Δ3a,EΔ2), (Δ3a,EΔ3), (Δ3a,EΔ4), (Δ3a,EΔ5), and (Δ3a,EΔ6)]; truncation of the E protein by introduction of a stop codon 6 aa upstream of the PBM [(Δ3a,E-ΔPBM)]; interruption of the E protein PBM by amino acid substitutions within its core sequence [(Δ3a,E-PBM^−^)]; and replacement of the E protein PBM with an alternative PBM sequence [(Δ3a,E-PBM*)]. The ΔPBM(rev) sequence represents the sequence of a spontaneous revertant virus isolated after growth of the original ΔPBM strain in cell culture. (B) Viability of SARS-CoV ΔE mutants with or without 3a protein PBM was evaluated in Vero E6 cells. Titers of the viable viruses after the first passage (representative of three replicate experiments) are shown. Data represent means ± SD. NR, not rescued.

To identify which E protein domain was essential in the absence of full-length 3a protein, a set of Δ3a mutants was engineered with substitutions or deletions throughout the E protein sequence (Fig. 10A), including (i) alanine substitutions in the N-terminal domain [rSARS-CoV-MA15-(Δ3a,EΔ1)]; (ii) disruption of the TMD, abrogating E protein IC activity [(Δ3a,E-N15A)] (34); (iii) short in-frame deletions throughout the C-terminal domain [(Δ3a,EΔ2), (Δ3a,EΔ3), (Δ3a,EΔ4), (Δ3a,EΔ5), and (Δ3a,EΔ6)]; (iv) truncation of E protein by the introduction of a stop codon 9 aa upstream of the PBM [(Δ3a,E-ΔPBM)]; (v) interruption of the E protein PBM by amino acid substitutions within its core sequence [(Δ3a,E-PBM^−^)]; and (vi) replacement of the E protein PBM with an alternative synthetic PBM sequence with proven binding to PDZ domains (46) [(Δ3a,E-PBM*)]. The viability of each mutant was determined after rescue from infectious bacterial artificial chromosome (BAC) clones. For each mutant, two independently generated BACs were analyzed (Fig. 10A). Mutations within the N-terminal domain or TMD of protein E did not affect virus viability, indicating that neither harbored the motif responsible for viroporin replacement. However, when the protein E PBM was disrupted by amino acid substitutions [(Δ3a,E-PBM^−^)], infectious virus was not rescued. Consistent with this, when the original PBM was substituted for an alternative one [(Δ3a,E-PBM*)], viable virus was recovered. Furthermore, the truncated mutant [(Δ3a,E-ΔPBM)] rapidly reverted in the first passage, losing the engineered stop codon during passage in cell culture and regaining the PBM [Δ3a,E-ΔPBM(rev)]. Collectively, these data strongly support the idea of the requirement of the PBM for viability of SARS-CoV (Fig. 10A) and indicate that it was the E protein with a functional PBM that compensated for the loss of the full-length 3a protein. An additional mutant was generated in which the protein 3a PBM was mutated in the context of full-length E protein deletion [(3a-PBM^−^,ΔE)], but the rescue of this virus was not possible (Fig. 10B), again showing the necessity of at least one of the two proteins with a functional PBM for virus viability in the context of complete deletion of either the E gene or the 3a gene.

Also, virus titers were significantly decreased after one passage for mutants Δ3a,EΔ1 and Δ3a,E-ΔPBM(rev) compared to the Δ3a virus (Fig. 10A). In addition, Δ3a viruses with small deletions throughout the C terminus of E protein were not viable, possibly due to the requirement of the 3a protein native structure or of a length essential for PBM availability for binding to other viral or cellular proteins (Fig. 10A). To analyze the relevance of protein shortening or folding, an additional mutant was constructed in the Δ3a background by filling in the EΔ4 deletion with an alanine-rich sequence in order to restore the original length or folding of the E protein [(Δ3a,EΔ4*)] (Fig. S2). This mutant virus was viable, indicating the lack of sequence specificity for the observed functionality. An alternative role for other domains located in the middle of the E protein C terminus cannot be completely ruled out.

10.1128/mBio.02325-17.2 FIG S2  Evaluation of the influence of the length of the E carboxy terminus on the replacement of the 3a protein. The viability of a SARS-CoV Δ3a mutant with a deletion of 6 amino acids in comparison to that of a reconstructed virus in which the deletion was filled with 3 of the 6 amino acids replaced by alanines was evaluated in Vero E6 cells. Download FIG S2, TIF file, 0.1 MB. Copyright © 2018 Castaño-Rodriguez et al. 2018 Castaño-Rodriguez et al. This content is distributed under the terms of the Creative Commons Attribution 4.0 International license.

**FIG 11:**
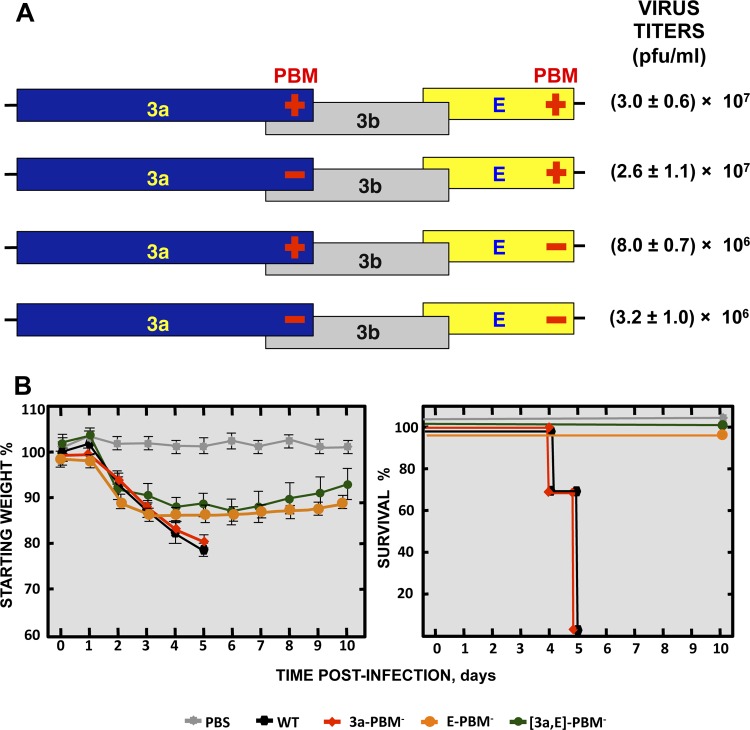
Virulence of recombinant SARS-CoV combining the knockdown of 3a and E protein PBMs. (A) Schematic of recombinant mutants with knockdown of the PBMs of the 3a and E proteins in different combinations. Titers of the viable viruses (representative of three replicate experiments) are shown. Data represent means ± SD. (B) Groups of five 16-week-old BALB/c mice were subjected to mock infection (PBS, gray filled squares) or infected with 100,000 PFU of the parental (wild-type) virus (WT; black filled hexagons) or of genetically engineered viruses lacking either the protein 3a PBM (3a-PBM^−^, red filled diamonds) or the E protein PBM (E-PBM^−^, ochre filled circles) or both [(3a,E)-PBM^−^, green filled circles]. Mean levels of weight loss (left) and survival (right) through 10 dpi are represented for each group. Data summarize two replicate experiments with equivalent results. Error bars represent the standard deviations of mouse weight data.

To further study replication dependence on the PBMs of E and 3a, a collection of mutants with one [(3a-PBM^−^) or (E-PBM^−^)] or both [(3a,E]-PBM^−^) viral PBM sequences removed was generated (Fig. 10A). All three PBM mutants were viable, showing that the consequences of the presence of E and 3a protein PBMs when these proteins have been completely deleted are different from those seen when only their PBM is missing. However, virus titers decreased 10-fold when both viral PBMs were missing in comparison to the levels seen with the parental virus or mutants lacking only one PBM (Fig. 11A). Remarkably, when PBM was present in E protein, the virus was highly virulent independently of whether the 3a protein included a PBM (Fig. 11B). In contrast, mortality significantly decreased when E protein lacked a PBM, regardless of the presence or absence of a PBM within protein 3a. These results indicated that the impact of the presence or absence of the E protein PBM on virulence is definitive, as it determines whether a virus is pathogenic or nonpathogenic, respectively. In contrast, the presence or absence of 3a protein with or without its PBM had little impact on virus virulence, illustrating the much greater relevance of the E protein PBM than of the 3a PBM with respect to virus pathogenicity.

## DISCUSSION

Viroporins are highly relevant for viral replication and pathogenesis, and their requirement in a large number of physiological processes makes their study a field of growing interest (17, 18, 23). CoVs usually encode two or more viroporins, including the conserved structural E protein and additional ones encoded by accessory genes. The roles of these viroporins in replication and virulence had been studied in detail only for SARS-CoV E protein (34, 46, 58, 60), though roles in replication have also been established for HCoV-OC43 ns12.9 (25), HCoV-229E 4a (24), and PEDV 3 (26). MERS-CoV encodes two proteins, E and 5, homologues of the SARS-CoV E and 3a proteins, respectively. While the IC activity of MERS-CoV E protein has been previously described (15), the potential IC activity of protein 5 is yet to be studied in detail. However, due to its similarity to SARS-CoV 3a, HCoV-229E 4a, and PEDV 3, IC conductance by this protein is expected. Furthermore, both the MERS-CoV E and 5 proteins have a putative PBM at their carboxy terminus, similarly to the SARS-CoV E and 3a proteins.

SARS-CoV encodes three viroporins: 3a, E, and 8a (14, 27, 28). We have previously shown that a SARS-CoV mutant lacking E protein was attenuated in mice (61). Here, we show that removal of the 3a protein slightly reduced virus titers *in vitro* compared to the results seen with the parental virus (Fig. 1A). In contrast, the titers of the Δ3a mutant were reduced 10-fold *in vivo* (Fig. 1B), indicating that the 3a protein was required for optimal SARS-CoV replication. This result is in agreement with previous studies showing a slight reduction of SARS-CoV-Δ3a titers in Vero E6 cells (62). Nevertheless, it has to be noted that the mice in the previous study were infected with a human SARS-CoV Urbani strain, which causes only a mild murine infection, in contrast to our mouse-adapted strain (61). The reduction of titers after infection with Δ3a mutant viruses may reflect the role of 3a in membrane rearrangement, increasing the levels of intracellular vesicles that can promote nonlytic release of viral particles (30). Alternatively, protein 3a may induce apoptosis (30, 63) and may enhance inflammation by activating nuclear factor kappa B (NF-kB), leading to the production of proinflammatory cytokines such as interleukin-8 (IL-8) and RANTES (CCL5) (29). In fact, histopathological analysis of lungs from SARS-CoV-Δ3a-infected mice showed minimal damage or cellular infiltration at 2 and 4 dpi, whereas mice infected with the SARS-CoV parental virus revealed interstitial and peribronchial cell infiltration and edema in both alveolar and bronchiolar airways at 2 dpi and, mainly, at 4 dpi (see Fig. S3 in the supplemental material). This suggests that the attenuation of the Δ3a mutant may be due to its inability to activate an exacerbated proinflammatory response, resulting in survival of infected mice. In fact, similar results were obtained in the lungs of mice infected with rSARS-CoV-MA15-ΔE, which also led to attenuation by downregulation of the host proinflammatory response (61, 64).

10.1128/mBio.02325-17.3 FIG S3  Lung pathology of mice infected with the SARS-CoV wt and Δ3a strains. Lung tissue sections from mice infected with wt or Δ3a viruses were prepared at 2 and 4 dpi and stained with hematoxylin and eosin. Three mice per group were analyzed independently. Download FIG S3, TIF file, 4.7 MB. Copyright © 2018 Castaño-Rodriguez et al. 2018 Castaño-Rodriguez et al. This content is distributed under the terms of the Creative Commons Attribution 4.0 International license.

We showed that deletion of SARS-CoV protein 8a alone did not have a measurable effect on replication and virulence in mice (Fig. 1 and 2). This is in line with the fact that ORF8 was lost during the SARS-CoV pandemic and that viruses lacking this gene were recovered from patients who died from SARS-CoV infection, supporting the idea that it is also not required for virulence in humans (65). However, viruses lacking 8a protein when 3a protein was also deleted had titers 10-fold lower than those seen with the parental virus, indicating that simultaneous deletion of the two proteins may contribute to replication to at least a small extent.

Residues involved in E protein IC activity have been previously identified (33), and this activity was essential for replication and virulence (34). We previously reported that the E ion channel activates the inflammasome through calcium release from intracellular stores (34, 58). IC activity of SARS-CoV E protein is exerted in the membrane of the ERGIC. This facilitates the release of Ca^2+^ from this intracellular compartment, which contributes to the activation of the inflammasome complex, leading to the release of proinflammatory cytokines such as tumor necrosis factor alpha (TNF-α), IL-1β, and IL-6. The accumulation of these cytokines promotes an exacerbated proinflammatory response, which leads to death. Thus, E protein IC activity is a virulence factor similar to the IC activities of M2 from influenza virus (19) or rotavirus NSP4 (13, 66). As E protein IC activity promotes virulence via inflammasome activation (34, 58), we hypothesized that protein 3a IC activity could act in a similar way. However, as no correlation was found between protein 3a IC activity and SARS-CoV titer and pathogenicity, we conclude that, in our mouse model, the IC activity of 3a protein did not affect replication and virulence in the same way as that of E protein. However, SARS-CoV 3a protein TMD3^−^ and TMD[2,3]^−^ mutants displayed no IC activity and were attenuated. In principle, a possible explanation for this observation is that the TMD3^−^ three-amino-acid mutations present in both mutant TMD3^−^ and mutant TMD[2,3]^−^ could have disrupted a function of the 3a protein other than its IC activity. Nevertheless, viruses that included the point mutations that disrupt IC activity (Y91A, H93A, and Y109A) and which are less likely to affect other functions of the 3a protein than the 3-amino-acid and 5-amino-acid mutations of TMD3^−^ and TMD[2,3]^−^, respectively, were completely virulent. The TMD3^−^ mutant showed 10-fold-lower titers in the lungs of infected mice. The impact of these mutants on virus replication and virulence may in principle be due to an exclusive effect on 3a protein functions different from its IC activity. The TMD[2,3]^−^ mutant maintained the same titers as the native virus, which could be explained if the mutations in TMD2 were structurally compensated for the ones in TMD3 when the two were simultaneously present in protein 3a. Overall, these results indicate that protein 3a IC activity is not involved in virus virulence.

There are several factors that may account for the differences in the degrees of relevance of the SARS-CoV E and protein 3a IC activities. The two proteins localize to different subcellular compartments (Fig. 8A), suggesting that the IC activity of the two proteins regulates ion transport between different compartments. E protein locates in the ERGIC (56) and induces calcium efflux during SARS-CoV infection, which activates the inflammasome complex, leading to the acute proinflammatory response associated with virus pathogenicity (34, 58). As protein 3a is not found at any of the main intracellular Ca^2+^ storage locations (Fig. 8B), its IC activity most likely induces cellular pathways that are less relevant to pathogenesis.

Note that SARS-CoV 3a protein subcellular localization had been previously studied by transfecting cells with a plasmid, which overexpresses a tagged variant of 3a protein, leading to the conclusion that protein 3a is located at the Golgi compartment or at the cell surface (31, 67). These studies have also been performed in the context of the infection but without the use of subcellular compartment markers, leading to the observation that protein 3a was located in the cell membrane, in the cytoplasm, and in the nucleus (27). After our analysis in the context of the infection using markers for cellular compartments, we could not conclude that the SARS-CoV 3a protein is located in any of the previously described compartments. Furthermore, knowledge of the cellular location in which the 3a protein accumulates remained elusive after our analysis performed using markers for the following different intracellular compartments: ER, Golgi apparatus, mitochondria, early and late endosomes, lysosomes, and the plasma membrane.

The E protein PBM is another virulence factor of SARS-CoV (46), with a homologous motif present in protein 3a. However, analysis of mutants lacking the 3a PBM showed no effect on virus production or virulence (Fig. 7). The requirement for protein E with its PBM was dominant for SARS-CoV virulence in comparison to the requirement for protein 3a with its PBM (Fig. 11). Furthermore, a SARS-CoV with E protein that included its PBM was virulent in the presence or absence of 3a protein PBM, whereas in the reverse situation (the presence of 3a protein with its PBM and of E protein lacking its PBM), the virus was always attenuated, reinforcing the idea of the dominance of the E protein PBM for virus pathogenicity over that of 3a protein.

The interaction of the E protein PBM with syntenin PDZ motifs activates the p38 mitogen-activated protein kinase (MAPK) pathway and promotes an acute proinflammatory response that leads to death (46). However, other viral PBMs likely show a preference for distinct PDZ domains. There are 266 motifs present in more than 400 cellular protein isoforms, each containing between 1 and 13 PDZ domains (42). As SARS-CoV E and 3a protein PBM sequences are different (DLLV and SVPL, respectively), they are likely to interact with different networks of PDZ-containing cellular proteins. The protein 3a PBM interaction with cellular PDZ proteins most likely induces a signaling pathway that either is not pathogenic for the host or is activated at a lower intensity due to a reduced affinity of the viral PBM for the cellular PDZ domain, explaining why viruses with or without the PBM of 3a showed no significant differences in pathogenicity. However, given that the protein 3a PBM is required for virus viability when full-length E protein is missing, it can be confirmed that the pathways activated by the protein 3a PBM have some impact on virus replication.

In order to study the interdependence of the SARS-CoV viroporins, the effects of deletion of one viroporin or of simultaneous deletions of two or three viroporins were determined. The 8a protein was included to determine its potential relevance in the absence of the other viroporins, showing a significant impact on viral growth under conditions in which 3a protein or E protein was absent. In contrast, we observed that SARS-CoV was not viable when both the 3a and E proteins were absent but that its viability was rescued by the presence of either 3a or E. We showed that the E protein could compensate for the loss of the other viroporin providing that it carried a functional PBM (Fig. 10A), a conclusion reinforced by the results obtained with five different recombinant viruses. Even more biologically relevant was the rapid reversion of a stop codon introduced 9 aa upstream of the carboxy terminus of the E protein in mutant ΔPBM. Reversion was identified only in this mutant, presumably because reversion of a single altered codon occurs more easily than the 4-aa changes in E-PBM^−^ or the deletions performed (Fig. 10A). Our results also showed that a 3a protein carrying a PBM could compensate for the loss of E protein and could restore virus replication but not virulence (Fig. 10B and 11).

The idea of the requirement of SARS-CoV PBMs for virus replication and virulence is also supported by our previous observations revealing that ΔE mutants evolved to introduce a new transmembrane protein with a PBM to compensate for the loss of the whole E protein during passage either in cell culture or *in vivo* (68). In addition, when SARS-CoV-ΔE was passaged in mice, it spontaneously gained an internal PBM in the 8a protein. PBMs are also phylogenetically conserved in proteins from SARS-CoV and MERS-CoV isolates from humans, civet cats, dromedary camels, and bats (Fig. S4), further reinforcing the idea of the relevance of PBMs for coronavirus viability. Viral PBM-cellular PDZ interactions have been previously described in several viruses. For instance, the PBM of E6 oncoprotein from human papillomavirus 16 (HPV-16) interacts with several cellular proteins containing PDZ motifs, leading to tumorigenesis and virus dissemination (69). On the other hand, different strains of rabies virus (RABV) include a variety of PBMs in the G protein that differentially contribute to viral virulence through activation of a variety of signaling pathways (70). In the case of influenza virus, NS1 protein has a PBM located at its carboxy terminus that is possibly involved in enhanced virulence (71). In addition, the vaccinia virus F11 protein has both a PDZ domain and a PBM, which influence viral spread (72).

10.1128/mBio.02325-17.4 FIG S4  PBMs conserved in SARS-CoV and MERS-CoV isolated from animals. The conserved PBMs in the carboxy terminus of SARS-CoV E and 3a proteins or MERS-CoV E and 5 proteins are shown. Download FIG S4, TIF file, 0.5 MB. Copyright © 2018 Castaño-Rodriguez et al. 2018 Castaño-Rodriguez et al. This content is distributed under the terms of the Creative Commons Attribution 4.0 International license.

Overall, we conclude that the SARS-CoV E and 3a proteins have in common two important characteristics: IC activity and PBM. Both the IC and the PBM of E protein are involved in SARS-CoV virulence and replication, whereas the corresponding motifs in protein 3a are not. We showed a dominance of E protein PBM and IC activities over that of the 3a protein homologues. However, the protein 3a PBM became relevant for virus viability in the absence of full-length E protein. These results contribute to a better understanding of the role of CoV IC activities and PBMs, with impacts on the rational design of future vaccines and antivirals.

## MATERIALS AND METHODS

### Ethics statement.

Animal experimental protocols were approved by the Ethical Committee of the Center for Animal Health Research (CISA-INIA) (permit numbers 2011-009 and 2011-09) in strict accordance with Spanish National Royal Decree (RD 53/2013) and international EU guidelines 2010/2063/UE and Spanish national law 32/2007. Infected mice were housed in a self-contained ventilated rack (Allentown, NJ).

### Viruses.

Mouse-adapted (MA15) (48) parental wild-type (wt) and recombinant viruses were rescued from infectious cDNA clones generated in a bacterial artificial chromosome (BAC) (49, 73–75).

### Generation of recombinant viruses.

Viruses with mutations in SARS-CoV viroporins E, 3a, and 8a were constructed in an infectious cDNA clone of SARS-CoV-MA15 within a bacterial artificial chromosome (BAC) (plasmid pBAC-SARS-CoV-MA15) (49, 73, 76). Generation of the E deletion mutant was described previously (61). The 3a gene was deleted by overlap extension PCR using pBAC-SARS-CoV-MA15 and the primers shown in Table S2 in the supplemental material. Mutations included deletion of a region (nt 25270 to 25668) of the SARS-CoV genome, resulting in deletion of the 3a protein while retaining the 3b protein; a disruption of the ATG start codon of the 3a gene; and point mutations at nt 25673 and 25683 to introduce two stop codons and a point mutation at nt 26042 to disrupt a potential initiation codon. A PCR product was generated from nt 24937 to 26060 of the SARS-CoV genome, digested at flanking SwaI and BamHI sites, and cloned into intermediate plasmid pBAC-SARS-*PmeI*-*BamHI*-SARS-CoV (which contains the nt-18404-to-26044 sequence of the SARS-CoV infectious cDNA clone) to generate plasmid pBAC-SARS-*PmeI*-*BamHI*-Δ3a. Finally, that plasmid was digested with PmeI and BamHI and the fragment carrying the 3a deletion was reinserted into a similarly digested pBAC-SARS-CoV-MA15 plasmid to generate pBAC-SARS-CoV-Δ3a. To construct the 8a deletion mutant, a DNA fragment containing nt 26790 to 28753 of the SARS-CoV genome flanked by restriction sites XcmI and NheI was assembled by overlap extension PCR using pBAC-SARS-CoV-MA15 as the template and the primers indicated in Table S1; this process resulted in the deletion of the first 82 nt of the 8a gene without affecting the 8b gene. The final PCR products were digested with XcmI and NheI and cloned into intermediate plasmid pBAC-*BamHI*-*RsrII*-SARS-CoV (which contains nt 26044 to 29782 of the SARS-CoV infectious cDNA clone [49]), thus obtaining pBAC-*BamHI*-*RsrII*-SARS-CoV-Δ8a. That plasmid was digested with BamHI and RsrII, and the fragment carrying the deletion of the 8a gene was reinserted into pBAC-SARS-CoV-MA15, generating pBAC-SARS-CoV-Δ8a.

To generate mutants with mutations in the 3a IC, DNA fragments containing nt 25016 to 26044 of the SARS-CoV genome were produced by overlap extension PCR using plasmid pBAC-SARS-CoV-MA15 as a template and the primers indicated in Table S1. The following mutations were introduced: S40A (TCA to GCA), S48A (AGC to GCC), Y91A (TAT to GCT), H93A (CAT to GCT), Y109A (TAT to GCT), Y113A (TAT to GCT), Q116A (CAA to GCA), TMD1^−^ (including both S40A and S48A), TMD2^−^ (including both Y91A and H93A), TMD3^−^ (including Y109A, Y113A, and Q116A), and TMD[2,3]^−^ (including Y91A, H93A, Y109A, Y113A, and Q116A). The PCR products were digested at flanking SwaI and BamHI sites and cloned into pBAC-*PmeI*-*BamHI*-SARS-CoV (49). Intermediate plasmids were digested with PmeI and BamHI and recloned into pBAC-SARS-CoV-MA15 to generate infectious SARS-CoV cDNA clones for each mutation. To mutate the PBM sequence of 3a, overlap extension PCR was performed using pBAC-SARS-CoV-MA15 as the template and the primers indicated in Table S2, resulting in a DNA fragment containing nt 26044 to 26790 of the SARS-CoV genome. The core 3a PBM (SVPL; nt AGCGTGCCTTTG), was replaced with an alternative sequence (GMSM; nt GGCATGTCTATG). As the 3a and 3b genes overlap in this region, care was taken to introduce only silent mutations into the 3b ORF. The resulting PCR fragment was digested at flanking BamHI and XcmI sites and cloned into intermediate plasmid pBAC-*BamHI-RsrII*-SARS-CoV (49), resulting in pBAC-*BamHI*-*RsrII*-SARS-CoV-3amutPBM. This plasmid was digested with BamHI and RsrII, and the fragment carrying the mutated 3a PBM was reinserted into pBAC-SARS-CoV-MA15 to generate plasmid pBAC-SARS-CoV-3a-PBM^−^.

10.1128/mBio.02325-17.6 TABLE S2  PCR primers used to generate SARS-CoV viroporin mutants. Download TABLE S2, DOCX file, 0.1 MB. Copyright © 2018 Castaño-Rodriguez et al. 2018 Castaño-Rodriguez et al. This content is distributed under the terms of the Creative Commons Attribution 4.0 International license.

Viruses combining deletion of the 3a gene with mutations throughout the E gene were generated by digesting previously described plasmids pBAC-SARS-CoV-MA15-EΔ1, -EΔ2, -EΔ3, -EΔ4, -EΔ5, and -EΔ6 (75) and -E-PBM^−^ and -E-PBM* (46) with BamHI and RsrII and cloning the fragments with mutations into a similarly digested pBAC-SARS-CoV-MA15-Δ3a plasmid.

In order to generate recombinant baculoviruses for 3a protein (rBV-3a), all constructs of the SARS-CoV 3a gene were cloned into a pFastBac vector (Invitrogen) containing a tobacco etch virus (TEV) cleavable site and 10 histidine residues fused to the C terminus of the 3a constructs. Recombinant baculoviruses were produced following the instructions of the manufacturer (Invitrogen).

### Recovery of recombinant SARS-CoV variants from the cDNA clones.

BHK cells were grown to 95% confluence in 12.5-cm^2^ flasks and transfected with 6 µg of infectious cDNA clone and 18 µl of Lipofectamine 2000 (Invitrogen), according to the manufacturer’s specifications. At 6 h posttransfection (hpt), cells were trypsinized, added to confluent Vero E6 cells monolayers grown in 12.5-cm^2^ flasks, and incubated at 37°C for 72 h. Cell supernatants were harvested and passaged once on fresh cells, and the recovered viruses were cloned by three rounds of plaque purification following standard procedures.

### Cells.

Vero E6 cells and BHK cells were kindly provided by E. Snijder (University of Leiden, the Netherlands) and H. Laude (Unité de Virologie et Immunologie Molecularies, INRA, France), respectively. In all cases, cells were grown in Dulbecco’s modified Eagle’s medium (DMEM; Gibco) supplemented with 25 mM HEPES, 2 mM l-glutamine (Sigma), 1% nonessential amino acids (Sigma), and 10% fetal bovine serum (FBS; BioWhittaker, Inc.). Virus titrations were performed in Vero E6 cells as previously described (61).

### Mice.

Eight-week-old specific-pathogen-free BALB/c Ola Hsd female mice were purchased from Harlan Laboratories and maintained for 8 additional weeks in the animal care facility at the National Center of Biotechnology (Madrid). For infection experiments, mice were anesthetized with isoflurane and intranasally inoculated at 16 weeks of age with 100,000 PFU of the indicated viruses. All work with infected animals was performed in a biosafety level 3+ (BSL3+) laboratory (CISA, INIA) by technicians wearing personal protection equipment (3M).

### Generation of polyclonal antibodies specific for the SARS-CoV 3a protein.

A synthetic peptide corresponding to residues 11 to 24 of the SARS-CoV 3a protein (C-ESITAQPVKIDNAS) was generated and used to immunize two rabbits (Biogenes, Berlin, Germany) according to the standard protocol of the supplier. Serums were collected at 45 dpi and evaluated by enzyme-linked immunosorbent assay (ELISA) and immunofluorescence and Western blot analysis using Vero E6 cells infected with SARS-CoV-wt or SARS-CoV-Δ3a as a negative control.

### Virus genome sequencing.

Regions of the SARS-CoV genome corresponding to the 3a, E, and 8a genes were sequenced after reverse transcriptase PCR (RT-PCR). Briefly, total RNA from infected cells or homogenized mouse lungs was collected and purified using an RNeasy kit (Qiagen) according to the manufacturer’s specifications. For RT reactions, 100 ng of RNA, random oligonucleotide primers, and ThermoScript reverse transcriptase (Invitrogen) were used. RT products were subsequently subjected to PCR using Vent polymerase (New England Biolabs) and the following primer pairs: 24937-VS (GGCGACATTTCAGGCATTAACGC) and 26086-RS (GGCACGCTAGTAGTCGTCGTCGGC), which amplify the 3a gene; E-VS (CTCTTCAGGAGTTGCTAATCCAGCAATGG) and E-RS (TCCAGGAGTTGTTTAAGCTTCTCAACGGTA), which amplify nucleotides 26017 to 26447, including the E gene; and 27545-VS (GGAGGTTCAACAAGAGCTCTACTCGCC) and 28008-RS (GACAGTTGATAGTAACATTAGGTGTGC), amplifying a region that includes the 8a gene. Sequence assembly and comparison with the parent consensus sequence were performed with SeqMan software (Lasergene, Madison, WI).

### Growth kinetics.

Subconfluent monolayers (90% confluence) of Vero E6 cells in 12.5-cm^2^ flasks were infected at a multiplicity of infection (MOI) of 0.001 with the indicated viruses. Culture supernatants were collected at 0, 4, 24, 48, and 72 h postinfection (hpi), and virus titers were determined as previously described (61). For the analysis of cell-associated virus, Vero E6 cells were infected at a MOI of 0.001 with the indicated viruses. At 24 and 48 hpi, cells were recovered in phosphate-buffered saline (PBS) buffer and disrupted by four freeze-thaw cycles. Samples were then centrifuged to remove cell debris, and supernatants were titrated as previously described (59).

### Virus infection and growth in mice.

BALB/c mice were anesthetized with isoflurane and intranasally inoculated with 100,000 PFU of virus mixed with 50 µl of DMEM. Weight loss and mortality were evaluated daily. To determine SARS-CoV titers, lungs were homogenized in PBS containing 100 IU/ml penicillin, 0.1 mg/ml streptomycin, 50 µg/ml gentamicin, and 0.5 µg/ml amphotericin B (Fungizone), using a gentleMACS dissociator (Miltenyi Biotec, Inc.). Virus titrations were performed in Vero E6 cells as previously described (61). Viral titers were expressed as PFU counts per gram of tissue.

### Histopathology.

Mice were sacrificed at 2 and 4 dpi. Lungs were removed, fixed in zinc formalin, and embedded in paraffin. Histopathological examinations were performed on sections stained with hematoxylin-eosin.

### Ion channel reconstitution and ionic current recording.

Planar bilayers were formed by apposition of two monolayers prepared from a mixture of 1,2-dioleoyl-*sn*-glycero-3-phosphocholine (DOPC), 1,2-dioleoyl-*sn*-glycero-3-phospho-l-serine (DOPS), and 1,2-dioleoyl-*sn*-glycero-3-phosphoethanolamine (DOPE) at a DOPC/DOPS/DOPE ratio of 3:1:1 (wt/wt) (Avanti Polar Lipids, Alabaster, AL) mixed in pentane at 5 mg/ml. Lipids were added on ~100-µm-diameter orifices in the 15-µm-thick Teflon partition that separated two identical chambers (50, 77); the orifices were pretreated with a 1% solution of hexadecane–pentane. Aqueous solutions of KCl, NaCl, or CaCl_2_ were buffered with 5 mM HEPES at pH 6. All measurements were performed at room temperature (23 ± 1°C). Ion channel insertion was achieved by adding 0.5 to 1 µl of a 300 µg/ml solution of recombinant protein in a buffer containing acetonitrile-isopropanol (40:60) on one side of the chamber (here referred to as the *cis* side).

An electric potential was applied using Ag/AgCl electrodes in 2 M KCl–1.5% agarose bridges assembled within standard 250-µl pipette tips. The potential was defined as positive when it was higher on the *cis* side, whereas the *trans* side was set to ground. An Axopatch 200B amplifier (Molecular Devices, Sunnyvale, CA) was used in the voltage-clamp mode to measure the current and the applied potential. The chamber and the head stage were isolated from external noise sources with a double metal screen (Amuneal Manufacturing Corp., Philadelphia, PA). Single-channel conductance data were obtained from current measurements under conditions of an applied potential of +100 mV and were evaluated using the Gaussian fit tool of Sigma plot 12 (Systat Software, Inc.).

The reversal potential, *E*_rev_, was determined as follows. First, a lipid membrane was formed at a given salt concentration gradient. Second, one or several channels were inserted into the bilayer and a net ionic current appeared due to the concentration gradient. Third, the ionic current through the channel or channels was manually set to zero by adjusting the applied potential. The potential needed to achieve zero current was then corrected using values corresponding to the liquid junction potentials of the electrode salt bridges (78) to obtain the final *E*_rev_.

### Confocal microscopy.

Vero E6 cells were grown to 90% confluence on glass coverslips and infected with wt SARS-CoV at a MOI of 0.3 PFU/cell. At 24 hpi, the medium was removed and cells were washed twice with PBS and fixed with 4% paraformaldehyde–PBS for 30 min at room temperature. Cells were subsequently washed twice with PBS, permeabilized for 10 min with ice-cold methanol, and then blocked with PBS containing 10% FBS for 40 min at room temperature. Immunofluorescence was performed using mouse Abs specific for SARS-CoV E protein (generated as described in reference 56) (1:500), protein disulfide isomerase (PDI; Abcam, Inc.) (1:500), 58 K (Abcam, Inc.) (1:100), aconitase 2 (Abcam, Inc.) (1:500), rab5 (BD Biosciences) (1:100), rab7 (BD Biosciences) (1:100), lamp-1 (Santa Cruz Biotechnologies) (1:50), and Na^+^/K^+^ ATPase (Santa Cruz Biotechnologies) (1:50) and rabbit Abs specific for SARS-CoV 3a protein (1:500). Primary antibodies were diluted in PBS containing 5% FBS and incubated for 90 min at room temperature, and then coverslips were washed four times with PBS before incubation with secondary antibodies was performed. Alexa 488- or Alexa 546-conjugated antibodies specific for the different species (Invitrogen) were diluted 1:500 in PBS containing 5% FBS and incubated for 45 min at room temperature. Nuclei were stained using DAPI (4′,6-diamidino-2-phenylindole; Sigma) (1:200), and coverslips were mounted in ProLong Gold anti-fade reagent (Invitrogen) and examined on a Leica SP5 confocal microscope (Leica Microsystems, Inc.). Image analysis was performed using ImageJ (79) and the JACoP plug-in (57). Three areas per image of 120 by 120 pixels with high accumulation of 3a protein were analyzed to determine Pearson’s coefficient (Pc). Pc values below 0.6 were considered negative for colocalization. Partial colocalization was considered to have occurred for Pc values between 0.6 and 0.85. Pc values between 0.85 and 1 were considered positive for colocalization.

### Production and purification of SARS-CoV 3a protein and its mutant variants from a recombinant baculovirus (rBV-3a).

H5 cells at 80% confluence were infected (MOI = 1) with rBV-3a (constructed as specified in the “Generation of recombinant viruses” section above) and incubated at 22°C for 72 h. Cells were harvested and resuspended in lysis buffer 1 (50 mM Tris-HCl, 300 mM NaCl, 0.5% Triton X-100, pH 7.5) supplemented with 1% protease inhibitor cocktail (Sigma). Protein extracts were centrifuged at 12,000 × *g* for 10 min at 4°C, and the pellets were resuspended in lysis buffer 2 (8 M urea, 50 mM Tris-HCl, 300 mM NaCl, 1% IGEPAL, 1 mM β-mercaptoethanol, 10 mM imidazole, pH 7.5). Each sample was sonicated three times for 20 s each time and centrifuged at 12,000 × *g* for 10 min at 4°C. Finally, the protein present in the supernatant was purified through metal affinity chromatography (IMAC) using cobalt resin (Clontech) following the manufacturer’s instructions. Every fraction from the purification process was analyzed in 12% polyacrylamide gels using Coomassie Blue EZBlue gel staining reagent (Sigma). Then, the obtained protein was desalted using a PD-10 desalting column (GE Healthcare) and eluted in PBS.

### Statistical analysis.

Two-tailed, unpaired Student’s *t* tests were used to analyze the differences in mean values between groups. All results were expressed as means ± standard deviations; *P* values of <0.1 were considered significant.
