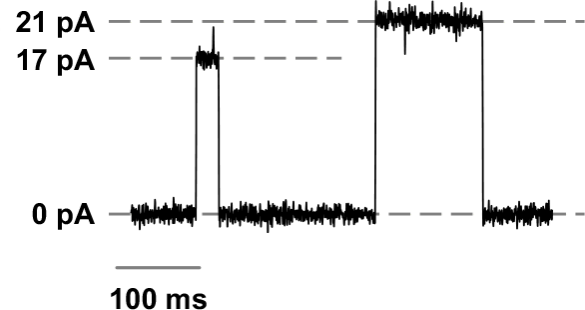# Correction for Castaño-Rodriguez et al., “Role of Severe Acute Respiratory Syndrome Coronavirus Viroporins E, 3a, and 8a in Replication and Pathogenesis”

**DOI:** 10.1128/mbio.02485-23

**Published:** 2023-11-10

**Authors:** Carlos Castaño-Rodríguez, Jose M. Honrubia, Javier Gutierrez-Álvarez, Marta L. DeDiego, Jose L. Nieto-Torres, Jose M. Jimenez-Guardeño, Jose A. Regla-Nava, Raul Fernandez-Delgado, Carmina Verdia-Báguena, Maria Queralt-Martín, Grazyna Kochan, Stanley Perlman, Vicente M. Aguilella, Isabel Sola, Luis Enjuanes

Volume 9, no. 3, e02325-17, 2018, https://doi.org/10.1128/mBio.02325-17. Page 6: Figure 3A should appear as shown in this correction. Regretfully we inadvertently swapped a sample current recording from the SARS-CoV-E protein and another current recording from the SARS-CoV-3a protein. The current values, though, are correct and the conclusions remain intact. We apologize for this error, which did not change the final result.

**Fig 3:**