# Supplementary figures and images for: Role of Severe Acute Respiratory Syndrome Coronavirus Viroporins E, 3a, and 8a in Replication and Pathogenesis
Source: mBio. 2018 May 22;9(3):e02325-17. doi: 10.1128/mBio.02325-17 (PMC5964350; doi:10.1128/mBio.02325-17)

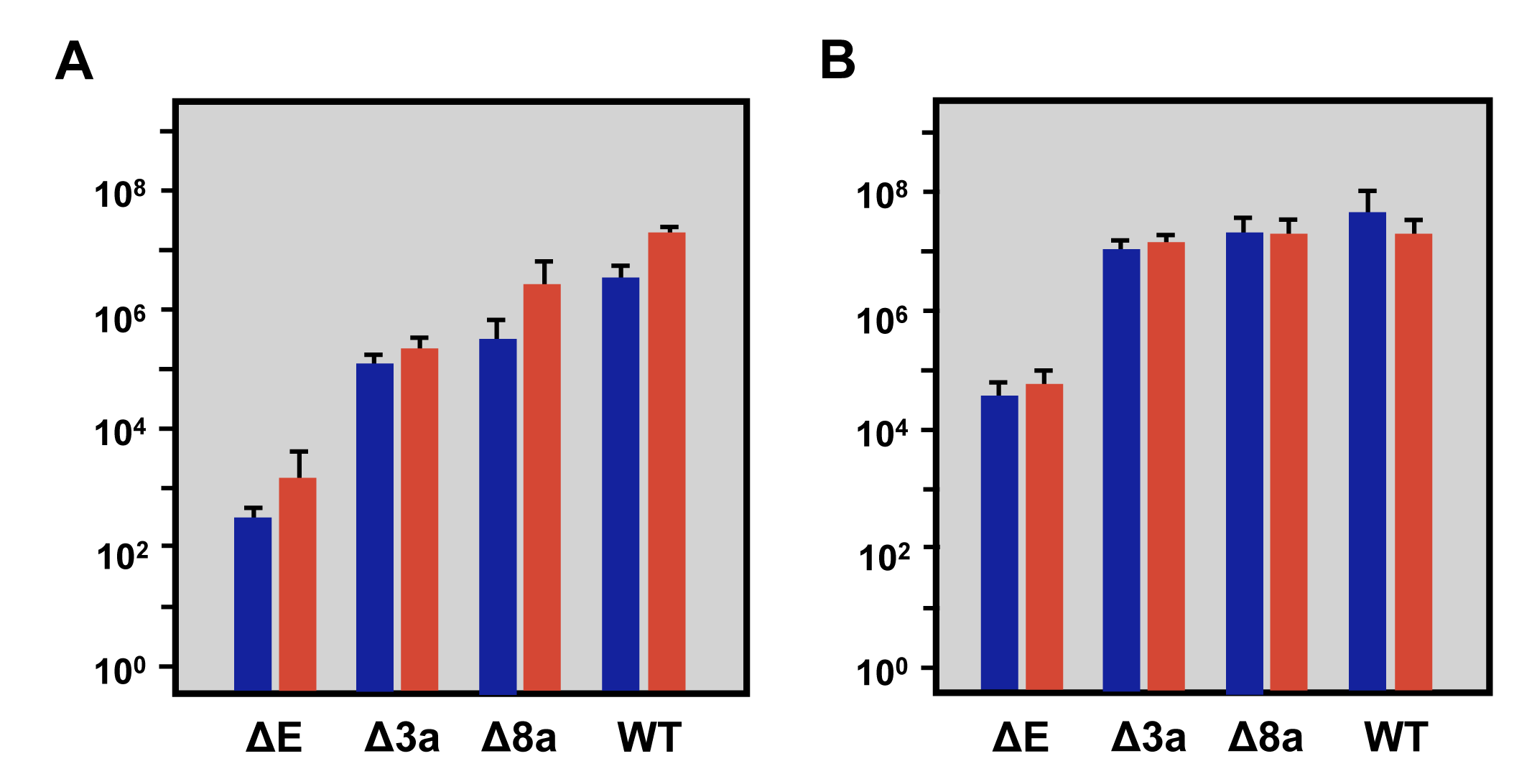

Supplement: FIG S1 [file mbo003183896sf1.tif]

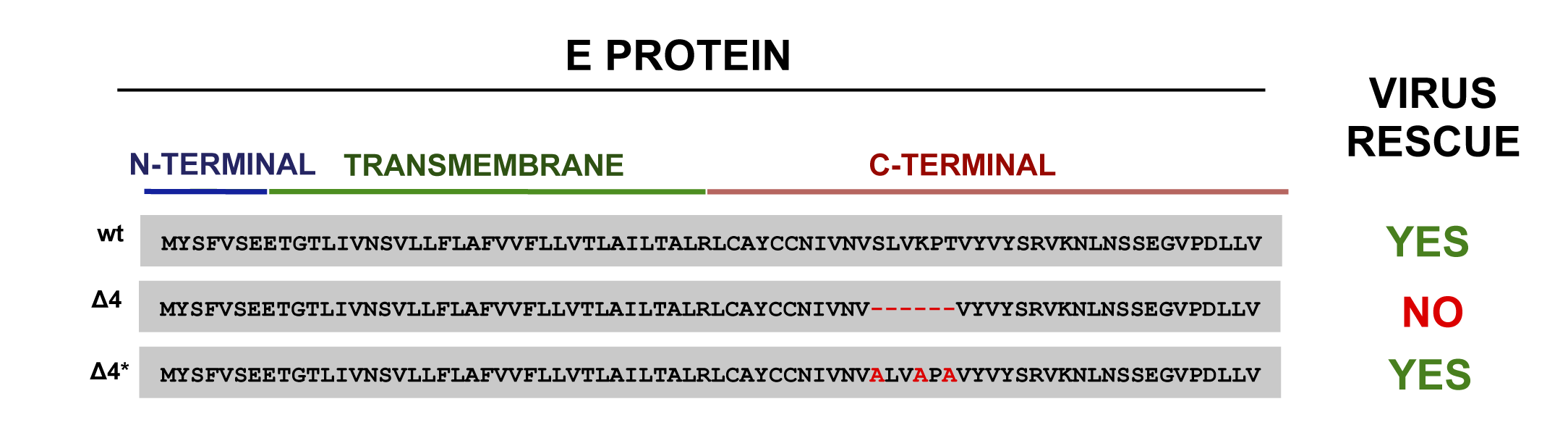

Supplement: FIG S2 [file mbo003183896sf2.tif]

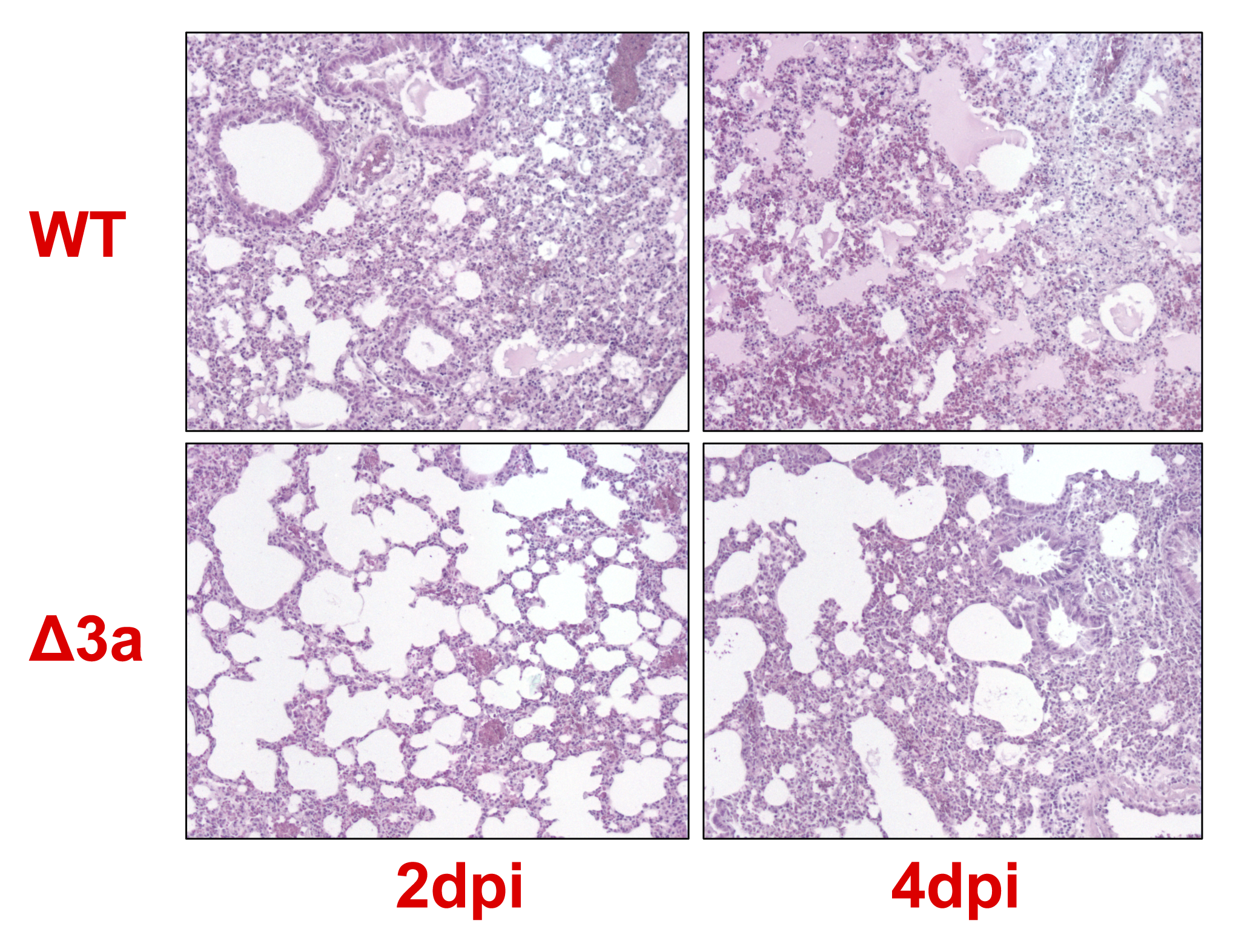

Supplement: FIG S3 [file mbo003183896sf3.tif]

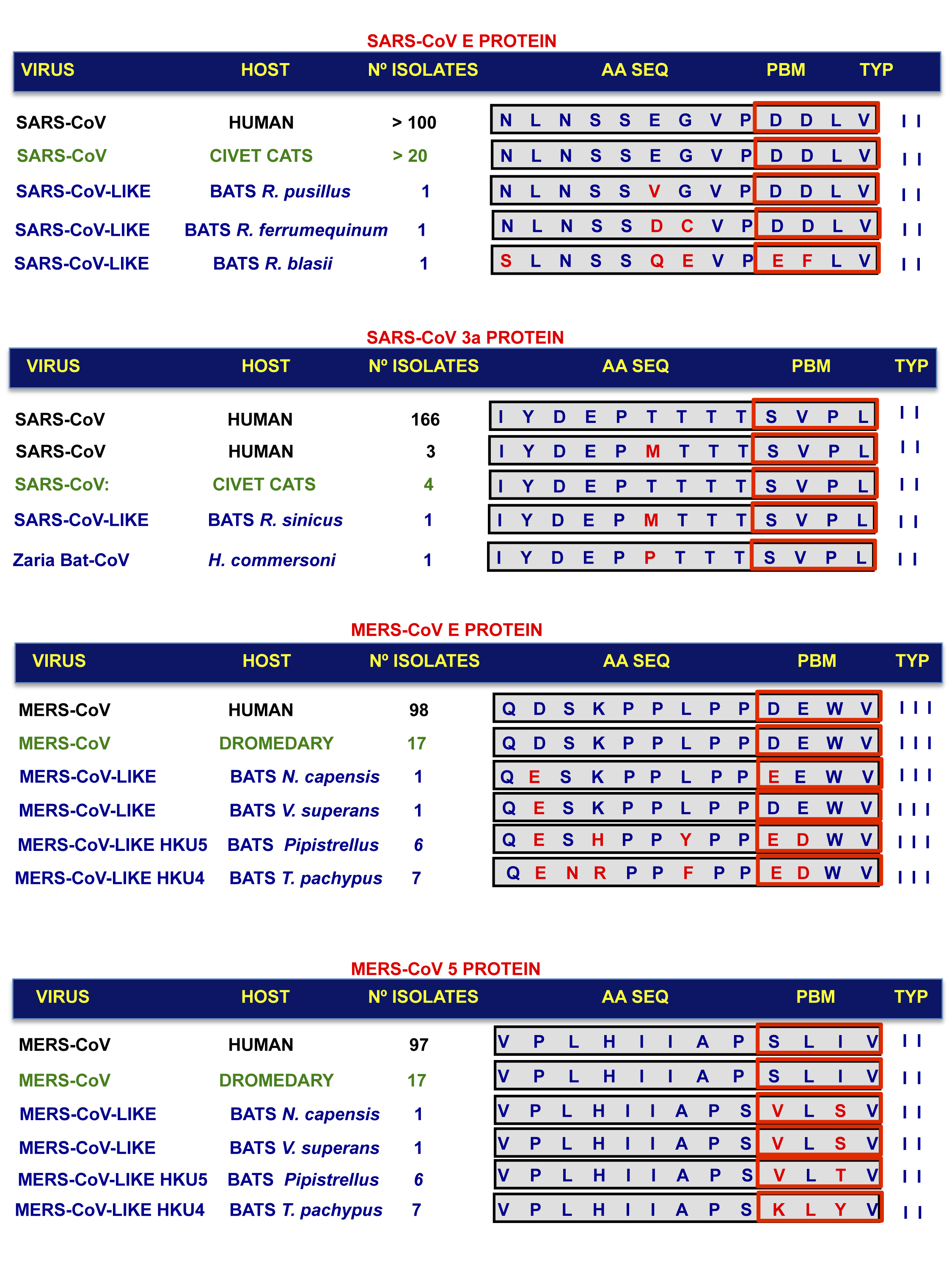

Supplement: FIG S4 [file mbo003183896sf4.tif]
