# Supplementary material for: Role of Severe Acute Respiratory Syndrome Coronavirus Viroporins E, 3a, and 8a in Replication and Pathogenesis
Source: mBio. 2018 May 22;9(3):e02325-17. doi: 10.1128/mBio.02325-17 (PMC5964350; doi:10.1128/mBio.02325-17)
Supplement: TABLE S1 [file mbo003183896st1.docx]

**Table S1. Pearson’s Coefficient analysis**

| Co-localization between | Pearson’s Coefficient |
| --- | --- |
| 3a protein & E protein | 0.24 |
| 3a protein & ER | 0.16 |
| 3a protein & Golgi | 0.17 |
| 3a protein & Mitochondria | -0.01 |
| 3a protein & Plasma Membrane | 0.04 |
| 3a protein & Early Endosomes | 0.07 |
| 3a protein & Late Endosomes | 0.47 |
| 3a protein & Lysosomes | -0.02 |

Pearson’s Coefficient above 0.6 are considered as positive co-localization.
