# Supplementary material for: Role of Severe Acute Respiratory Syndrome Coronavirus Viroporins E, 3a, and 8a in Replication and Pathogenesis
Source: mBio. 2018 May 22;9(3):e02325-17. doi: 10.1128/mBio.02325-17 (PMC5964350; doi:10.1128/mBio.02325-17)
Supplement: TABLE S2 [file mbo003183896st2.docx]

**Table S2. PCR primers used to generate SARS-CoV viroporin mutants.**

| **mutant*** | **PCR** | **Primer** | **Sequence** |
| --- | --- | --- | --- |
| ∆3a | 1  2 | SARS-24937-VS  SARS-25235-∆3a-RS  SARS-25261-∆3a-VS  SARS-26060-∆3a-RS | GGCGACATTTCAGGCATTAACGC CATCATAAAGT**T**ATGGGTTCT**A**GGAT**T**ATAAGTTCGTTTATGTGTAATGTAATTTGACACCC CGAACTTAT**A**ATC**T**AGAACCCAT**A**ACTTTATGATGCC  CATCATAAATTGGATCCA**C**TGCTGGATTAGCAACTCCTG |
| ∆8a | 1  2 | SARS-26788-VS  SARS-∆8a-RS  SARS-∆8a-VS  SARS-28757-RS | CCGGGGGACAATTGTGACCAGAC GGATCTTCAAGCACATGTTCGTTTAGACTTTGGTACAAGGTTCTTCTAGATCC  CCAAAGTCTAAACGAACATGTGCTTGAAGATCCTTGTAAGGTACAACACTAGG  GGGCAGTTTCACCACCTCCGCTAGC |
| TMD1**^-^** | 1  2 | SARS-24936-VS  SARS_3a_TMD1-_RS  SARS_3a_TMD1-_VS  SARS-26086-RS | GGCGACATTTCAGGCATTAACGCTTCTGTCG  CG**GC**CTGAAAAACAGCAAGAAATGCAACGCCAATAACAAGCCATCCGAAAGGGAGTG**C**GGCTTGTAGCGGTATC  C**G**CACTCCCTTTCGGATGGCTTGTTATTGGCGTTGCATTTCTTGCTGTTTTTCAG**GC**CGCTACCAAAATAAT  ggcacgctagtagtcgtcgtcggc |
| TMD2**^-^** | 1  2 | SARS-24936-VS  SARS_3a_TMD2-_RS  SARS-25546-VS  SARS-26086-RS | GGCGACATTTCAGGCATTAACGCTTCTGTCG  CAAAAATTGCGCCTCCATACCTGCAGCGACAAGCAAAAGA**GC**TGAA**GC**GATGGTAACAAATAGCAGC  TCTTTTGCTTGTCGCTGCAGGTATGGAGGCGCAATTTTTG  ggcacgctagtagtcgtcgtcggc |
| TMD3**^-^** | 1  2 | SARS-24936-VS  SARS-25586-RS  SARS-TMD3-VS  SARS-26086-RS | GGCGACATTTCAGGCATTAACGCTTCTGTCG  CAAAAATTGCGCCTCCATACCTGCAGCGACAAGCAAAAGA  TCTTTTGCTTGTCGCTGCAGGTATGGAGGCGCAATTTTTGTACCTC**GC**TGCCTTGATA**GC**TTTTCTA**GC**ATGCATCAACGCATGTAGA  ggcacgctagtagtcgtcgtcggc |
| TMD[2,3]**^-^** | 1  2 | SARS-24936-VS  SARS-TMD2-RS  SARS-TMD3-VS  SARS-26086-RS | GGCGACATTTCAGGCATTAACGCTTCTGTCG  CAAAAATTGCGCCTCCATACCTGCAGCGACAAGCAAAAGA**GC**TGAA**GC**GATGGTAACAAATAGCAGC  TCTTTTGCTTGTCGCTGCAGGTATGGAGGCGCAATTTTTGTACCTC**GC**TGCCTTGATA**GC**TTTTCTA**GC**ATGCATCAACGCATGTAGA  ggcacgctagtagtcgtcgtcggc |
| 3a-Y91A | 1  2 | SARS-24936-VS  SARS-3a-Y91A-RS  SARS-25546-VS  SARS-26086-RS | GGCGACATTTCAGGCATTAACGCTTCTGTCG  CAAAAATTGCGCCTCCATACCTGCAGCGACAAGCAAAAGATGTGAA**GC**GATGGTAACAAATAGCAGC  TCTTTTGCTTGTCGCTGCAGGTATGGAGGCGCAATTTTTG  ggcacgctagtagtcgtcgtcggc |
| 3a-H93A | 1  2 | SARS-24936-VS  SARS-3a-H93A-RS  SARS-25546-VS  SARS-26086-RS | GGCGACATTTCAGGCATTAACGCTTCTGTCG  CAAAAATTGCGCCTCCATACCTGCAGCGACAAGCAAAAGA**GC**TGAATAGATGGTAACAAATAGCAGC  TCTTTTGCTTGTCGCTGCAGGTATGGAGGCGCAATTTTTG  ggcacgctagtagtcgtcgtcggc |
| 3a-Y109A | 1  2 | SARS-24936-VS  SARS-25586-RS  SARS-Y109A-VS  SARS-26086-RS | GGCGACATTTCAGGCATTAACGCTTCTGTCG  CAAAAATTGCGCCTCCATACCTGCAGCGACAAGCAAAAGA  TCTTTTGCTTGTCGCTGCAGGTATGGAGGCGCAATTTTTGTACCTC**GC**TGCCTTGATATATTTTCTACAATGCATCAACGCATGTAGA  ggcacgctagtagtcgtcgtcggc |
| 3a-Y113A | 1  2 | SARS-24936-VS  SARS-25586-RS  SARS-Y113A-VS  SARS-26086-RS | GGCGACATTTCAGGCATTAACGCTTCTGTCG  CAAAAATTGCGCCTCCATACCTGCAGCGACAAGCAAAAGA  TCTTTTGCTTGTCGCTGCAGGTATGGAGGCGCAATTTTTGTACCTCTATGCCTTGATA**GC**TTTTCTACAATGCATCAACGCATGTAGA  ggcacgctagtagtcgtcgtcggc |
| 3a-Q116A | 1  2 | SARS-24936-VS  SARS-25586-RS  SARS-Q116A-VS  SARS-26086-RS | GGCGACATTTCAGGCATTAACGCTTCTGTCG  CAAAAATTGCGCCTCCATACCTGCAGCGACAAGCAAAAGA  TCTTTTGCTTGTCGCTGCAGGTATGGAGGCGCAATTTTTGTACCTCTATGCCTTGATATATTTTCTA**GC**ATGCATCAACGCATGTAGA  ggcacgctagtagtcgtcgtcggc |
| 3a-PBM^-^ | 1 | SARS-3amutPBM-VS  SARS-26885-RS | GGATCCAATTTATGATGAGCCGACGACGACTACT**G**GC**A**TG**T**CT**A**TGTAAGCACAAGAAAGTGAGTA  GGTCCTTAATGTCACAGCGCCC |

The introduced mutations are shown in bold.
